# Dietary Blueberry Supplementation Attenuates the Effects of an Ultra‐Processed Food Cafeteria Diet on Weight Gain and Metabolic Parameters, Enhancing Nutrigenomic Profiles in C57BL/6 Mice

**DOI:** 10.1002/mnfr.70206

**Published:** 2025-08-22

**Authors:** Felipe Mateus Pellenz, Mayara Souza de Oliveira, Shiva Cerutti Witteé, Joana Raquel Nunes Lemos, Bianca Gomes Panis, Isadora Cristina Godoy de Lima, Celina Fagundes da Silva, Anna Carolina Meireles Vieira, Mariana Rauback Aubin, Luciane Moretto, Monique Banik Siqueira, Eliandra Girardi, Raif Gregorio Nasre‐Nasser, Karla Suzana Moresco, Letícia de Almeida Brondani, Guilherme Coutinho Kullmann Duarte, Rafael Aguiar Marschner, Bianca Marmontel de Souza, Daisy Crispim

**Affiliations:** ^1^ Endocrine Division Hospital De Clínicas De Porto Alegre Porto Alegre Rio Grande do Sul Brazil; ^2^ Faculdade de Medicina Post‐Graduate Program in Medical Sciences: Endocrinology Universidade Federal do Rio Grande Do Sul Porto Alegre Rio Grande do Sul Brazil; ^3^ Postdoctoral Researcher in Diabetes Research Institute and Clinical Cell Transplant Program University of Miami Miller School of Medicine Miami Florida USA; ^4^ Departamento De Nutrição Universidade Federal do Paraná Paraná Brazil; ^5^ Unit of Laboratorial Research, Experimental Research Center Hospital De Clínicas de Porto Alegre Porto Alegre Rio Grande do Sul Brazil

**Keywords:** blueberry, cafeteria diet, hepatic steatosis, insulin resistance, obesity, supplementation

## Abstract

The cafeteria diet (CAFD) model mimics Western dietary patterns, inducing obesity in mice. Blueberry (BB) consumption improves metabolic outcomes due to its anti‐inflammatory and antioxidant properties, though mechanisms remain unclear. This study assessed BB supplementation effects on biometric, metabolic, and hepatic steatosis parameters in CAFD‐fed mice, and analyzed obesity‐related gene expression in adipose tissues, liver, muscle, and hypothalamus. Thirty‐two male C57BL/6 mice were divided into three groups: Control (C, standard diet—SD; *N* = 10), CAF (CAFD + SD; *N* = 12), and BB (SD + CAFD + BB; *N* = 10). BB animals received 22.4 g of freeze‐dried BB per week. After 16 weeks, biometric and glycemic parameters, insulin resistance (IR), hepatic steatosis, oxidative stress markers, and serum leptin, adiponectin, and irisin levels were evaluated. Expression of genes related to apoptosis, lipid and glucose metabolism, oxidative stress, and adipocytokine pathways was analyzed by qPCR. BB supplementation improved biometric, glycemic, IR, hepatic steatosis, and oxidative stress and antioxidant markers compared to CAF. Leptin, adiponectin, and irisin levels decreased in BB mice. Also, BB consumption modulated the expression of obesity‐related genes. BB mitigated CAFD‐induced weight gain, IR, hepatic steatosis, oxidative stress, and obesity‐related gene dysregulation, highlighting its nutrigenomic potential.

## Graphical abstract

1. Experimental Design (left panel)

∘ Three C57BL/6 mice groups

∘ Control group (Standard chow)

∘ Cafeteria diet (cookies, soda, sausage, condensed milk, chow)

∘ CAFD + Blueberry (CAFD + BB smoothie and pellets)

** • Group Labels**:

∘ **CAFD**: Cafeteria diet + SD

∘ **BB**: CAFD + BB

** • Duration**: 16 weeks

• Arrow pointing to next panel

2. Key Findings (center panel)

∘ CAFD **→** ↑ weight, ↑ Lee index

∘ BB **→** prevented obesity

∘ CAFD **→** fatty liver

∘ BB **→** prevented fatty liver

∘ CAFD **→** ↑ adiponectin, irisin and leptin

∘ BB → prevented adipokines upregulation

3. Key Findings (left panel)

• Glucose & Insulin Resistance

∘ CAFD **→** ↑ glucose, insulin, and HOMA‐IR

∘ BB **→** partially prevented insulin resistance

• Oxidative stress and Antioxidant status

∘ CAFD **→** ↑ oxidative stress ↓ antioxidant status

∘ BB **→** ↓ oxidative stress ↑ antioxidant status

• Nutrigenomics

∘ **BB modulated**:

∘ ↑ anti‐inflammatory/apoptosis‐protective genes (*Adipoq*, *Glut4*, *Ppargc1a*, *Bcl2*)

∘ ↓ pro‐inflammatory genes (e.g., *Itgax*, *Il1b*, *Nlrp3*, *Casp1*)

4. Conclusion (at bottom left)

**Blueberry supplementation reduces obesity, improves metabolic parameters, and restores obesity‐related gene expression, highlighting its nutrigenomic potential in the context of an ultra‐processed cafeteria diet**.

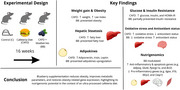

**Abbreviations**
AT: adipose tissue
BAT: brown adipose tissue
BB: blueberry
CAFD: cafeteria diet
DIO: diet‐induced obesity
GSH: glutathione
GST: glutathione‐S‐transferase
HFD: high‐fat diet
HOMA‐IR: homeostatic model assessment of insulin resistance
IR: insulin resistance
KEGG: Kyoto Encyclopedia of Genes and Genomes
OGTT: oral glucose tolerance test
ROS: reactive oxygen species
SAT: subcutaneous adipose tissue
SD: standard diet
SOD: superoxide dismutase
T2DM: type 2 diabetes mellitus
TBARS: thiobarbituric acid
UPFs: ultra‐processed foods
VAT: visceral adipose tissue

## 1 Introduction

Obesity is a chronic and multifaceted disease characterized by abnormal or excessive fat accumulation in the body that may impair health [1]. Currently, up to 2 billion individuals worldwide are affected by excessive body weight, making this disease an epidemic of the 21st century [2]. Obesity is intricately linked to multiple comorbidities, such as cardiometabolic disorders, type 2 diabetes mellitus (T2DM), nonalcoholic liver disease, and certain types of cancer [2, 3, 4]. Notably, the adipose tissue (AT) of affected individuals undergoes adaptive processes, including adipocyte hyperplasia and hypertrophy, which lead to the recruitment of macrophages that polarize toward pro‐inflammatory states [5, 6]. Additionally, the enlargement of the AT contributes to the increased release of free fatty acids, reactive oxygen species (ROS), and pro‐inflammatory cytokines, which, in turn, contribute to the low‐grade chronic inflammation observed in individuals with obesity [5, 6].

The pathogenesis of obesity is multifactorial, influenced by genetic, epigenetic, endocrine, neurological, behavioral, sociocultural, and environmental factors [7, 8, 9]. Advances in genomic technologies have allowed the identification of multiple genetic loci associated with obesity, primarily involved in regulating body weight, food intake, and satiety [9, 10]. Over 1000 genetic variants have been associated with obesity and its related traits [11]. Although the heritability of body mass index (BMI) ranges between 40% and 70% [12], environmental triggers, such as physical inactivity and dietary patterns influenced by societal changes, undeniably modulate body fat distribution [13].

The consumption of ultra‐processed foods (UPFs) has increased in parallel to the incidence of obesity [14]. Commonly referred to as junk foods, UPFs are energy‐dense, low in fiber, and high in sugar, salt, and saturated fatty acids, making them nutritionally unbalanced products [14]. The cafeteria diet (CAFD) model for animal experiments has been validated as a preclinical model of obesity, as it mimics the metabolic effects of dietary choices typical of Western societies [15, 16]. The CAFD model features a combination of foods with different textures, nutrients, and tastes, including highly palatable salty, fatty, and sweet products [14, 16]. Consequently, the CAFD provides a robust animal model for inducing obesity and related‐metabolic complications and low‐grade inflammation compared to other diet‐induced obesity models [16, 17].

Recently, food‐derived bioactive compounds, such as polyphenols and anthocyanins, have been highlighted as regulators against several chronic diseases [18]. These nutrients exhibit antioxidative and anti‐inflammatory properties, protecting cells and tissues from oxidative damage [18, 19, 20]. Among berries, blueberries (BB; *Vaccinium* spp.) have the most diverse profile of anthocyanins [19, 20]. Notably, regular BB intake has been associated with reduced risk of cardiovascular disease, mortality, T2DM, and improved weight maintenance in humans [19, 20, 21]. In animal models of diet‐induced obesity (DIO), particularly those using a high‐fat diet (HFD), a number of studies have reported benefits of BB consumption in glucose and lipid homeostasis, as well as favorable impacts on anti‐inflammatory parameters [19, 22, 23, 24, 25, 26]. However, to our knowledge, no previous study has evaluated the impact of BB consumption within the context of the CAFD model.

Thus, our study aimed to investigate the influence of BB consumption on biometric, glycemic, insulin resistance (IR), hepatic steatosis, adipocytokine, and oxidative stress outcomes in C57BL/6 mice fed a CAFD compared to chow‐fed mice. Furthermore, we assessed the effect of BB supplementation on the expression of genes related to apoptotic, lipid and glucose metabolism, oxidative stress, and adipocytokine pathways in subcutaneous AT (SAT), visceral AT (VAT), brown AT (BAT), liver, skeletal muscle, and hypothalamus from these animals. To the best of our knowledge, this is the first study to evaluate the impact of BB consumption on obesity‐related metabolic outcomes within the context of a CAFD in C57BL/6 mice.

## 2 Experimental Section

### 2.1 Experimental Design and Ethics Statement

This experimental study was designed and conducted following the ARRIVE guidelines for animal research [27, 28]. Thirty‐two male C57BL/6 mice (45 days old; weight mean ± standard deviation: 19.8 ± 2.0 g) were included. The animals were housed two per cage in the Animal Experimentation Unit of Hospital de Clínicas de Porto Alegre (HCPA). Mice were maintained under conditions of a 12‐h light/dark cycle at a temperature of 22°C ± 2°C with controlled humidity (40%–60%), and an air exhaustion system. The study protocol (DIPE‐HCPA number 2019‐0546) received approval from the Institutional Ethical Committee on Animal Use of the HCPA and adhered to the established procedures for the scientific use of animals.

### 2.2 Dosage Information

**FIGURE 1:**
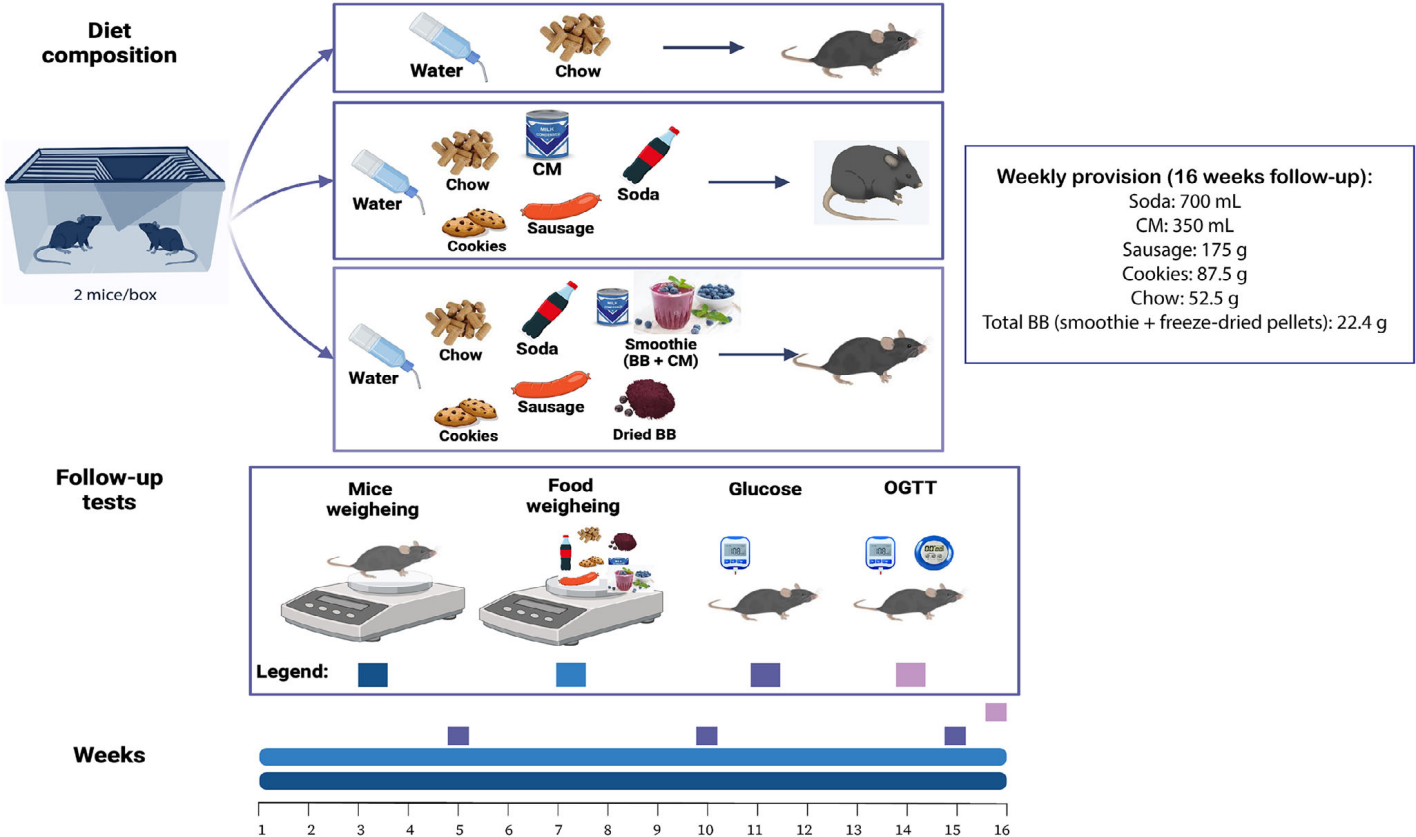
Diet composition and experimental design during the 16‐week follow‐up period. Mice were weighed once a week, and food weighing was performed every 2 days for up to 16 weeks. Blood glucose levels were measured monthly after 6 h of fasting, while the OGTT was performed in the last week after 6 h of fasting. *n* = 10 in C, *n* = 12 in CAF, and *n* = 10 in BB, representing biological replicates. BB = blueberry, C = control, CAF = cafeteria diet, CM = condensed milk, OGTT = oral glucose tolerance test.

C57BL/6 mice were randomly assigned to three experimental groups: (1) the control group (C; *n* = 10), which was fed only a standard diet (SD); (2) the CAF group (*n* = 12), which was exposed to the CAFD; and (3) the BB group (*n* = 10), which was fed a combination of CAFD and freeze‐dried BB. The CAF and BB groups also received the SD to avoid nutritional deficiencies. All groups were followed up for 16 weeks, with their weight and glucose levels recorded, as shown in Figure 1. Of note, CAFD and BB supplementations were administered simultaneously for a period of 16 weeks, which encompassed the entire duration of the experimental protocol. Moreover, the C group received only the SD (chow) and water ad libitum. They were housed in a separate room from the CAF and BB groups to prevent any interference related to the smell of the provided diets.

The CAFD composition was designed to closely replicate human consumption patterns, being hypercaloric and rich in processed carbohydrates and saturated fatty acids compared to the SD diet. The CAFD diet protocol was adapted from Estadella et al. [29], and its composition closely resembles the Western diet, presenting high palatability, energy‐dense foods, and increased sodium content. This dietary profile is expected to induce low‐grade inflammation, a characteristic often observed in patients with obesity [30]. All nutritional values for CAF and BB groups were calculated and expressed per 100 g of diet to allow for a standardized comparison across groups, as shown in Table 1.

**TABLE 1:** Nutritional composition of cafeteria diet offered during the 16‐week follow‐up.

Composition	Control^*^ Nuvilab CR‐1 (NUVITAL) (100 g)	Cafeteria diet (100 g)	Cafeteria diet + blueberries (100 g)
Energy, kcal	293.0	184.3	178.5
kcal	2.93	1.84	1.78
Protein, g (%)	17.72 (24.2)	5.02 (10.9)	5.60 (13.0)
Carbohydrate, g (%)	31.81 (65.9)	25.3 (55)	24.6 (57.0)
Total sugar, g	NP	17.3	14.2
Total fat, g (%)	3.22 (9.9)	6.98 (34.1)	5.9 (30.0)
Saturated fatty acids, g	NP	3.3	2.1
Trans fatty acids, g	NP	0.15	0.12
Polyunsaturated fatty acids, g	NP	0.33	0.25
Fiber, g	NP	0.23	0.30
Cholesterol, mg	NP	19.52	17.30
Sodium, mg	NP	185	162

*Note*: Values equivalent to a 100 g portion.

Abbreviations: kcal = kilocalories, NP = not provided.

* Values for the control group were provided by the product manufacturer. For the cafeteria diet and cafeteria diet + blueberry groups: Nutritional values were calculated based on commercial product information.

For the CAF group, the weekly provision per housing box included 700 mL of coke soda, 350 mL of condensed milk (both provided using standard rodent water bottles with stainless steel sipper tubes), 175 g of sausage, 87.5 g of cookies, and 52.5 g of SD to prevent nutritional deficiencies. The nutritional composition of CAFD included 55% carbohydrates, 34.1% total fat (24% from saturated fatty acids), 10.9% proteins, 185 mg sodium, and 19.52 mg cholesterol, totaling 1.84 kcal/g, as shown in Table 1.

For the BB group, in addition to the CAFD and SD components described above, animals also received 22.4 g of freeze‐dried BB per week, which was offered in two forms and replaced every 2 days: a smoothie made of 3.2 g of freeze‐dried BB blended with 10 mL of condensed milk, and 40 mL of water (provided using standard rodent water bottles with stainless steel sipper tubes), and 3.2 g of solid freeze‐dried BB pellets. Importantly, no additional condensed milk, beyond that provided in the CAFD, was provided to this group, as the amount used in the smoothie served solely as a vehicle for BB delivery. The selection of the BB supplementation method (smoothie and freeze‐dried pellets) was based on the results of a pilot experiment, as detailed in Supplementary Material 1. The nutritional composition of the BB group comprised 57% carbohydrates, 30% total fat (35.6% from saturated fatty acids), 13% proteins, 162 mg sodium, and 17.30 mg cholesterol, totaling 1.78 kcal/g (Table 1).

The dose of BB supplementation was determined based on a literature systematic review publication [19], which reported that dietary BB concentrations in experimental studies typically range from 0.5% to 10% (wt/wt). We selected the upper limit of 10% (wt/wt) relative to total food intake, equivalent to 12 g of fresh BB per day or 24 g every 2 days, considering two mice per cage. To account for an estimated 40% food loss [19], the provision was increased to 32 g of fresh BB per cage every 2 days. Since the BB was administered in freeze‐dried form, and freeze‐drying typically retains approximately 20% of the original fresh weight [19], the dose was adjusted to 6.4 g of freeze‐dried BB per cage every 2 days. This amount was delivered into pellets (3.2 g) and a smoothie (3.2 g) prepared with condensed milk and water. Based on these calculations, BB supplementation accounted for approximately 15% of the animals’ total daily dietary energy intake, representing 21.54 kcal. When extrapolated to humans, 15% of a standard 2000 kcal/day corresponds to approximately 300 kcal, which could be achieved through the consumption of about 82 g of freeze‐dried BB supplements.

For the C group, the animals received Nuvilab CR‐1 (NUVITAL), the standard rodent chow, consisting of 65.9% carbohydrates, 9.9% total fat, 24.2% proteins, and other constituents such as vitamins and fibers, totaling 2.93 kcal/g (according to the manufacturer's information). Of note, all experimental groups received water and SD ad libitum. Moreover, all foods were replaced with fresh items every 2 days and were freely available.

### 2.3 Monitoring of Diet Composition, Body Weight, and Lee Index

Food consumption was monitored every 2 days over a 16‐week period to assess the dietary intake across all groups. Intake was determined by calculating the difference between the amount of food provided and the leftover food in each housing cage. Consumption was evaluated using two approaches: (1) by analyzing the intake of each specific food item, and (2) by evaluating the total meal consumption, expressed as the average intake per housing box. This allowed for the calculation of macro‐ and micronutrient ingestion. Additionally, we conducted weekly weight measurements of each animal, and the delta (Δ) weight was calculated using the following equation: final weight − initial weight.

The Lee index (g/cm^3^) is commonly used as an equivalent to the BMI in humans and can indicate whether a rodent is lean or obese [31]. To determine the Lee Index, the naso‐anal length (cm) of each mouse was measured. The index is calculated using the formula: the cube root of the body weight (g) divided by the naso‐anal length (cm), multiplied by 1000 [31].

### 2.4 Evaluation of Blood Glucose, Oral Glucose Tolerance Test (OGTT), and Insulin Resistance

Capillary glycemia was evaluated by collecting blood through puncture with a 30G needle in the lateral tail vein. The Freestyle Optium Neo H Glucometer (Abbott, Chicago, USA) was used for this measurement, requiring a sample volume of 6.0 µL. This monitoring occurred monthly following a 6‐h fasting period, with water provided ad libitum.

The oral glucose tolerance test (OGTT) was performed during the last week of the follow‐up period after a 6‐h fasting period, with water available ad libitum. Blood glucose levels were measured at 0, 30, 60, 90, and 120 min post‐ingestion of a 2 g/kg glucose solution, administered directly into the stomach of each mouse using a gavage probe. Blood samples were collected by puncturing the lateral tail vein with a 30G needle at 0 (before glucose administration) and at 30, 60, 90, and 120 min after glucose administration. The Freestyle Optium Neo H Glucometer was used for these measurements, with a sample volume of 6.0 µL.

Insulin levels were determined using an ELISA immunoassay (Rat/Mouse Insulin, ELISA EZRMI‐13K, Sigma‐Aldrich, St. Louis, MO, USA; assay range: 0.2–10 ng/mL). IR was estimated using the homeostatic model assessment (HOMA) index, calculated with the following formula: fasting serum insulin levels (µg/L) × fasting plasma glucose levels (mg/dL)/405 [32, 33]. Adiponectin, leptin, and irisin levels in the serum of fasted mice were quantified using ELISA kits (Adiponectin: KMP004 [assay range: 0.125–8 ng/mL] and Leptin: KMC2281 [assay range: 93.8–6.000 pg/mL], Thermo Fisher Scientific, Waltham, MA, USA, and Irisin: DY9420‐05, DuoSet, R&D Systems, Bio‐Techne, USA; assay range: 76.0–800 pg/mL).

### 2.5 Sample Collection

In the last week of follow‐up, the animals underwent a 6‐h overnight fast and were subsequently anesthetized using inhaled isoflurane. Euthanasia was performed via cardiac puncture, and blood was collected in tubes containing separating gel. Serum was separated by centrifugation at 2000 × *g* for 10 min at 15°C and stored at −80°C. BAT, SAT, VAT, hypothalamus, liver, and skeletal muscle were isolated, washed with ice‐cold 50 mM phosphate buffer saline (pH 7.4), patted dry, placed in RNALater solution (Thermo Fisher Scientific), and stored at −80°C for subsequent gene expression analyses. Moreover, a portion of the liver was fixed in buffered 10% formaldehyde (pH 7.4) for histological analysis. The brain and another portion of the liver were stored at −80°C for assessment of oxidative stress parameters and antioxidant status.

### 2.6 Measurement of Oxidative Stress and Antioxidant Markers in Brain and Liver

Brain and liver tissue homogenates were prepared by homogenizing 100 mg of tissue in a 20 mM sodium phosphate buffer (pH 7.4) containing 140 mM KCl, using a tissue homogenizer. The homogenates were then centrifuged at 800 × *g* [34], and the resulting supernatants were used for the analysis related to oxidative stress and antioxidant markers.

Regarding oxidative stress parameters, carbonyls were measured according to Zanatta et al. [35]. Samples were treated with 2,4‐dinitrophenylhydrazine, and absorbance was read at 370 nm. Data were calculated using the millimolar absorption coefficient of hydrazine (∑370 nm = 21 000 M^−1^ cm^−1^), and results are described as nmol carbonyl/mg protein [35, 36]. Thiobarbituric acid reactive species (TBARS) content was assessed following the method described by Yagi et al. [37]. A calibration curve was created using 1,1,3,3‐tetramethoxypropane. Reactions were read at excitation and emission wavelengths of 515 and 553 nm, respectively. Results are expressed as nmol of TBARS/mg protein [36, 37]. Sulfhydryl content was measured according to Aksenov and Markesbery [38]. Samples were treated with 5,5’‐dithiobis(2‐nitrobenzoic acid), and absorbance was determined at 412 nm. Results are expressed as nmol sulfhydryl/mg protein [36, 38].

Considering antioxidant markers, glutathione (GSH) concentration was assessed according to Browne and Armstrong [39]. A calibration curve was prepared with standard GSH (0.001–1 mM), and the reactions were exposed to wavelengths of 350 and 420 nm (excitation and emission, respectively). Concentrations are reported as nmol of GSH/mg protein [36, 39]. Glutathione‐S‐transferase (GST) activity was assessed following the method of Carlberg and Mannervick [40]. GST activity was determined by monitoring NADPH consumption at 340 nm. Results are expressed as U/mg of protein [36, 40]. Superoxide dismutase (SOD) activity was measured according to Marklund et al. [41]. A calibration curve was established using purified SOD as a standard, and absorbance was measured at 420 nm. Results are described as U/mg of protein [36, 41].

### 2.7 RNA Isolation and Quantitative Real‐Time Polymerase Chain Reaction (qPCR)

Total RNA was extracted from 50–60 mg of BAT, SAT, VAT, hypothalamus, and liver using the PureLink RNA Mini Kit (Thermo Fisher Scientific) according to the manufacturer's protocol. Additionally, total RNA was extracted from 50 mg of skeletal muscle using the TRIzol RNA Isolation Reagent (Thermo Fisher Scientific) as per the manufacturer's instructions. The RNA concentration and quality of each sample were assessed using a NanoDrop One spectrophotometer (Thermo Fisher Scientific), and only samples that met the required quality criteria were used in further analyses. Reverse transcription of 200–800 ng of RNA into cDNA was carried out using the High‐Capacity cDNA Reverse Transcription Kit (Thermo Fisher Scientific) following the manufacturer's protocol, with concentrations depending on each tissue and gene analyzed.

cDNA was amplified using the quantitative real‐time PCR (qPCR) technique, performed by monitoring the real‐time fluorescent increase of PowerUP SYBR Green Master Mix (Thermo Fisher Scientific) [42]. Specific primers for 36 genes were designed using published mouse sequences and Primer Express 3.0 software (Thermo Fisher Scientific). Table S1 provides the sequence information for all the primers used. These genes were selected from the following obesity‐related Kyoto Encyclopedia of Genes and Genomes (KEGG) pathways [43], using *Mus musculus* as the species filter: adipocytokine signaling (pathway ID: mmu04920), lipid and atherosclerosis (mmu05417), NOD‐like receptor signaling (mmu04621), insulin resistance (mmu04931), glucagon signaling (mmu04922), thermogenesis (mmu04714), apoptosis (mmu04210), MAPK signaling (mmu04010), and HIF‐1 signaling (mmu04066) pathways. In VAT, we also evaluated the expression of markers for distinct macrophage and monocyte phenotypes: *Llgl1* (a marker of M2/MGL1+ resident macrophages), *Itgax* (a marker of pathogenic M1/CD11c+ polarized macrophages), and *Lgals3* (Galectin 3+, a marker of monocyte recruitment to VAT).

The qPCR reactions were performed using the ViiA 7 RT‐PCR system (Thermo Fisher Scientific). The relative expression of each gene in the six tissues of interest was determined using the comparative ΔΔCq method [44, 45], with results expressed relative to the respective reference gene. Table S2 provides details on cDNA concentrations and the reference gene used for each target. Validation assays were conducted by independently amplifying the target and the reference genes through serial dilutions of a cDNA sample. Both target and reference genes showed consistent amplification efficiencies (*E* = 95% to 105%) in all experiments, meeting the method criteria. qPCR specificity was confirmed through melting curve analyses, which demonstrated that all primers generated amplicons with a single, sharp peak during the amplification.

### 2.8 Statistical Analysis

Variables are presented as mean ± standard error of the mean (SEM). The normality of quantitative variable distributions was evaluated through the Kolmogorov–Smirnov and Shapiro–Wilk tests. Variables that did not follow a normal distribution were log‐transformed before analysis. Quantitative variables were compared between the C, CAF, and BB groups using one‐way ANOVA or Kruskal–Wallis (when log‐transformation did not normalize the distributions) tests. Generalized estimating equation (GEE) tests were used to compare cumulative energy intake, Δ‐weight, glucose measurements, and OGTT results among the groups over the follow‐up period. Furthermore, correlations between gene and protein expressions and laboratory and biometric parameters were assessed using Spearman correlation tests. Statistical significance was set at *p* < 0.05, and all analyses were performed using SPSS version 18 (SPSS Inc., Chicago, IL, USA). Graphs were created using GraphPad Prism 5 (Dotmatics, MA, USA). Of note, only statistically significant differences (*p* < 0.05) are described in the text of the different subsections of the results. mplete data are shown in tables and figures.

## 3 Results

### 3.1 Food Intake During Follow‐Up Period

The total weight of food intake over the follow‐up period showed an increase in both the CAF and BB groups. In the CAF group, food intake per mouse increased from 85.34 to 92.01 g by the 15th week. Interestingly, in the BB group, total food intake per mouse ranged from 55.67 g at baseline to 84.95 g by the 15th week. Regarding total calorie intake, the CAF group showed a range from 105.43 kcal per mouse at baseline to 101.00 kcal by the 15th week (Figure S1). Similarly, in the BB group, total calorie intake ranged from 109.77 kcal per mouse at baseline to 116.77 kcal per mouse in the 15th week (Figure S1). Throughout the 16‐week follow‐up period, diet and nutrition composition were maintained, resulting in a mean intake of 46.33% carbohydrates, 43.55% lipids, and 10.12% proteins for the CAF group, and a mean intake of 51.89% carbohydrates, 39.78% lipids, and 8.33% proteins for the BB group.

### 3.2 BB Consumption Prevents Weight Gain and Size Increase

**FIGURE 2:**
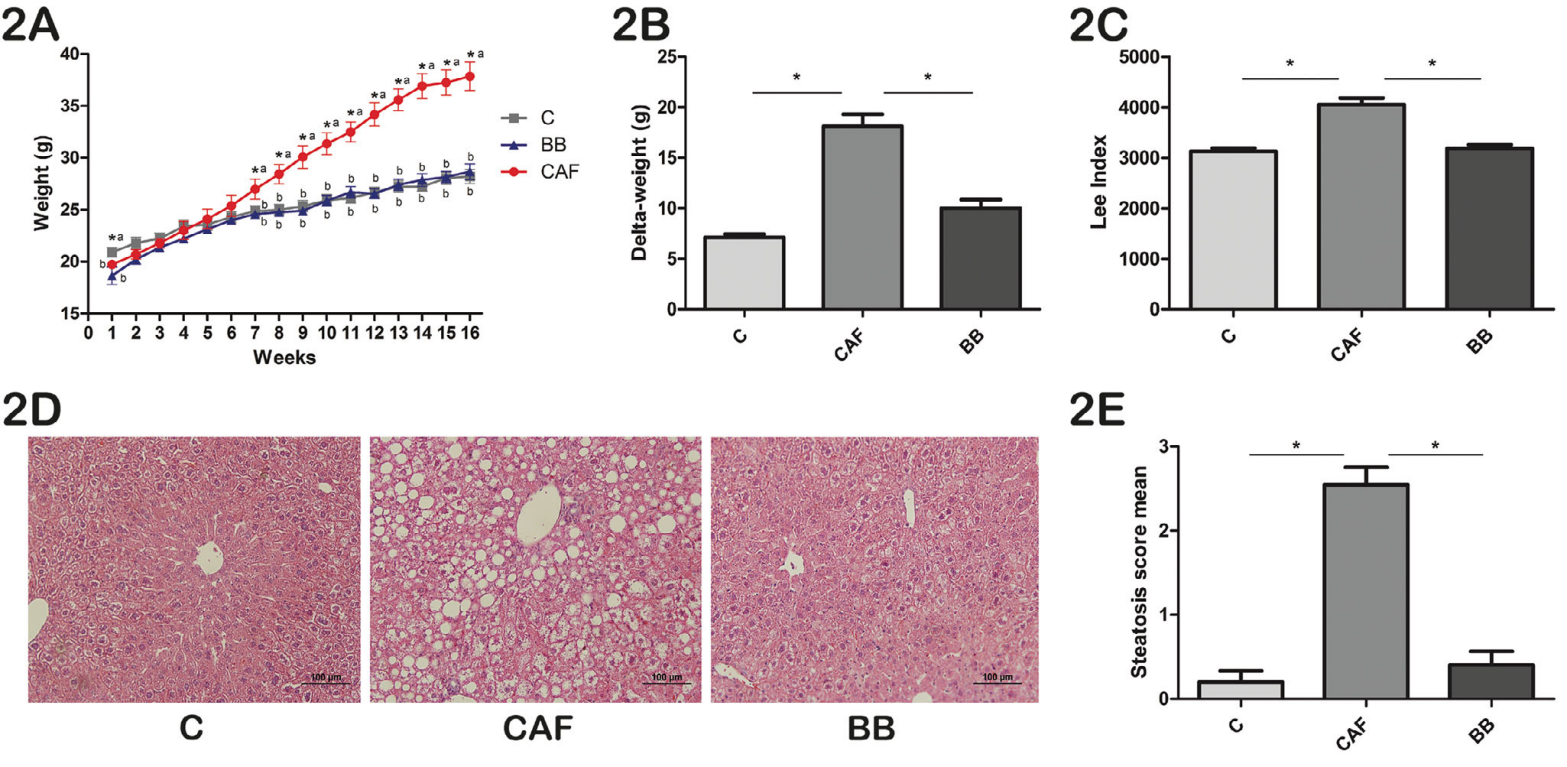
BB consumption prevents weight gain, obesity, and nonalcoholic hepatic steatosis after 16 weeks of follow‐up. (A) Body weight gain during the 16‐week follow‐up. (B) Delta‐weight. (C) Lee Index. (D) Liver histological sections stained with hematoxylin‐eosin (200×). Images are representative of one C, one CAF, and one BB mouse. (E) Mean hepatic steatosis score. Values are expressed as mean ± SEM. *n* = 10 in C, *n* = 11 in CAF, and *n* = 10 in BB, representing biological replicates. Significant differences are indicated by ^*^ *p* < 0.05 according to GEE (A) or one‐way ANOVA followed by post hoc (B, C, and E) tests. For comparisons in panel (A): variables with equal letters did not differ significantly in the GEE, and those with different letters were statistically different. BB = blueberry, C = control, CAF = cafeteria diet.

Body weight was slightly higher in the C group compared to both the CAF and BB groups at baseline (Table 2). Mice fed the CAFD significantly increased their weight after 7 weeks and continued gaining weight until the end of the experiment (Figure 2A; Table 2). Notably, mice in the BB group had similar weights to those in the C group at the end of the 16‐week follow‐up (Figure 2A; Table 2). Consequently, the Δ‐weight of the BB group was lower than the CAF group, but did not differ from the C group (Figure 2B; Table 2). Moreover, BB consumption prevented size increase, which also directly affected the Lee index values (Table 2). CAF mice had higher Lee index values compared to the BB and C groups at the end of the 16‐week follow‐up period (Figure 2C; Table 2).

**TABLE 2:** Biometric and metabolic data at the end of the 16‐week follow‐up.

Variables	C (*n* = 10)	CAF (*n* = 12)	BB (*n* = 10)	p**
Body weight (g)—baseline	20.9 ± 0.46^a^	19.7 ± 0.40^ab^	18.6 ± 0.84^b^	0.040
Body weight (g)^*^	28.2 ± 0.67^a^	37.8 ± 1.39^b^	28.7 ± 0.75^a^	< 0.0001
Δ‐weight (g)^*^	7.1 ± 0.32^a^	18.1 ± 1.18^b^	10.0 ± 0.84^a^	< 0.0001
Size (cm)^*^	9.0 ± 0.13^a^	9.33 ± 0.09^b^	9.0 ± 0.07^a^	0.025
Lee index (g/cm^3^)^*^	312.7 ± 6.39^a^	405.1 ± 13.40^b^	318.7 ± 7.00^a^	< 0.0001
Fasting glucose (mg/dL)^*^	123.9 ± 4.83^a^	186.1 ± 8.40^b^	154.1 ± 8.46^c^	< 0.0001
Circulating insulin levels (ng/mL)^*^	0.88 ± 0.12^a^	4.92 ± 1.38^b^	2.28 ± 0.35^c^	< 0.0001
HOMA‐IR^*^	0.27 ± 0.04^a^	2.26 ± 0.65^b^	0.85 ± 0.12^c^	< 0.0001

*Note*: Values are expressed as mean ± SEM.

Abbreviations: BB = cafeteria diet + blueberries, C = control (standard diet), CAF = cafeteria diet, HOMA‐IR = homeostasis model assessment of insulin resistance index.

* Values for Week 16. *p* values were obtained with one‐way ANOVA followed by a post hoc test. ** Variables with equal letters did not differ significantly in the statistical tests, and those with different letters were statistically different.

### 3.3 BB Protects Against Hepatic Steatosis and Influences Insulin Sensibility and Glucose Homeostasis

**FIGURE 3:**
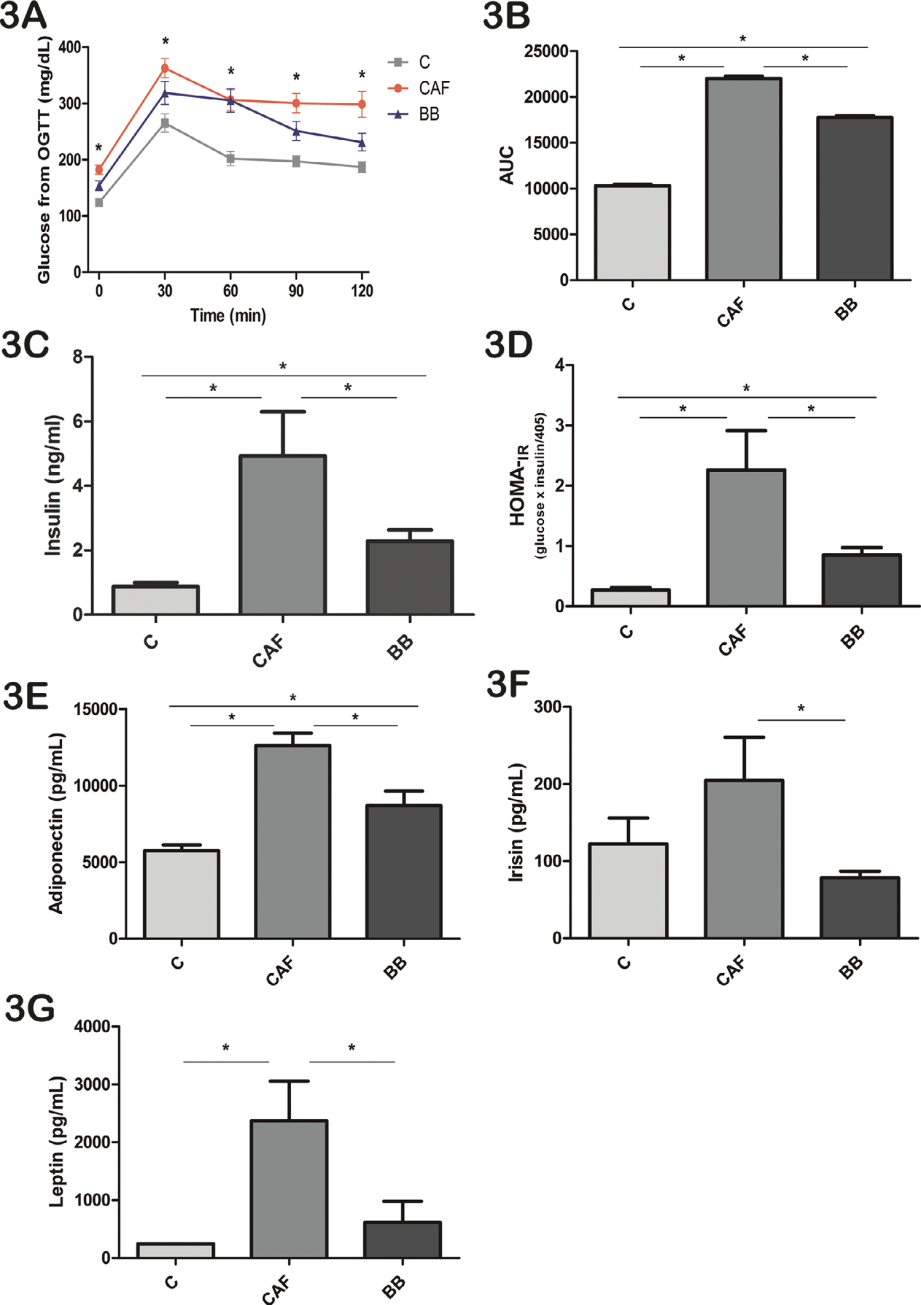
Influence of BB supplementation on glucose homeostasis, insulin resistance, and fasting adiponectin, irisin, and leptin circulating levels. (A) Fasting glucose (mg/dL) during the OGTT. (B) AUC calculated from the OGTT. (C) Fasting insulin levels (ng/mL). (D) HOMA‐IR index. (E) Fasting adiponectin levels (pg/mL). (F) Fasting irisin levels (pg/mL). (G) Fasting leptin levels (pg/mL). Values are expressed as mean ± SEM. *n* = 10 in C, *n* = 11 in CAF, and *n* = 10 in BB, representing biological replicates. Significant differences are indicated by * *p* < 0.05. According to GEE (A) or one‐way ANOVA followed by post hoc (B)–(G) tests. For comparisons in panel (A): variables with equal letters did not differ significantly in the GEE, and those with different letters were statistically different. AUC = area under the curve, BB = blueberry, C = control, CAF = cafeteria diet, HOMA‐IR = homeostasis model assessment of insulin resistance index, OGTT = oral glucose tolerance test.

BB consumption prevented CAFD‐induced nonalcoholic fatty liver as demonstrated by the lower hepatic steatosis score at 16 weeks compared to the CAF group, being similar to those values observed in the C mice (Figures 2D–E). At the end of the 16th week, BB consumption was able to partially decrease fasting blood glucose levels compared to the CAF group (Table 2). During the OGTT, CAF mice exhibited the highest peak of glucose levels at 30 min post‐glucose ingestion compared to the C and BB groups (Figure 3A). Consistently, the area under the curve (AUC) of the OGTT was higher in the CAF group compared to the C group (Figure 3B). BB consumption partially decreased the AUC of the OGTT (Figure 3B). Insulin levels were also elevated in the CAF group compared to both C and BB animals (Figure 3C; Table 2). Additionally, the homeostasis model assessment of insulin resistance (HOMA‐IR) index (Figure 3D; Table 2) and adiponectin levels (Figure 3E) were at intermediate values in the BB group compared to CAF and C mice. Irisin circulating levels were decreased in BB animals compared to CAF mice (Figure 3F), and leptin levels were markedly decreased in both the C and BB mice compared to the CAF group (Figure 3G).

### 3.4 BB Intake Influences Oxidative Stress and Antioxidant Markers in Brain and Liver

**FIGURE 4:**
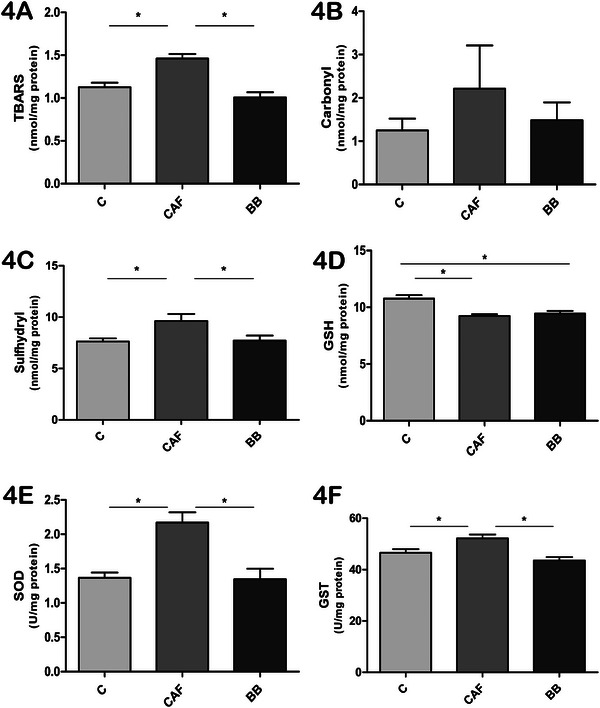
BB supplementation influences oxidative stress and antioxidant markers in the brain. (A) TBARS levels (nmol/mg protein). (B) Carbonyl levels (nmol/mg protein). (C) Sulfhydryl content (nmol/mg protein). (D) GSH concentration (nmol/mg protein). (E) SOD activity (U/mg protein). (F) GST activity (U/mg protein). Values are expressed as mean ± SEM. *n* = 10 in C, *n* = 11 in CAF, and *n* = 10 in BB, representing biological replicates. Significant differences are indicated by ^*^ *p* < 0.05 according to one‐way ANOVA followed by post hoc tests. BB = blueberry, C = control, CAF = cafeteria diet, GSH = glutathione, GST = glutathione‐S‐transferase, SOD = superoxide dismutase, TBARS = thiobarbituric acid reactive species.

Next, we evaluated oxidative stress and antioxidant markers in the brain and liver of the mice. Descriptive results for each assessed marker are presented in Table S3. Protein and lipid oxidation markers in the brain and liver were assessed through carbonyl and TBARS measurements, respectively. In the brain, TBARS levels were significantly increased in the CAF group compared to both the C and BB groups (Figure 4A), while carbonyl levels showed no significant differences between the groups (Figure 4B). Interestingly, sulfhydryl content, which are molecules involved in regulation of antioxidant and catalysis reactions, were higher in the CAF group compared to the other two groups (Figure 4C). The concentration of the free radical scavenger GSH was decreased in both the CAF and BB animals compared to the C group (Figure 4D). Regarding antioxidant enzymes, SOD and GST activities were higher in the CAF group compared to the C and BB groups (Figures 4E and 4F).

**FIGURE 5:**
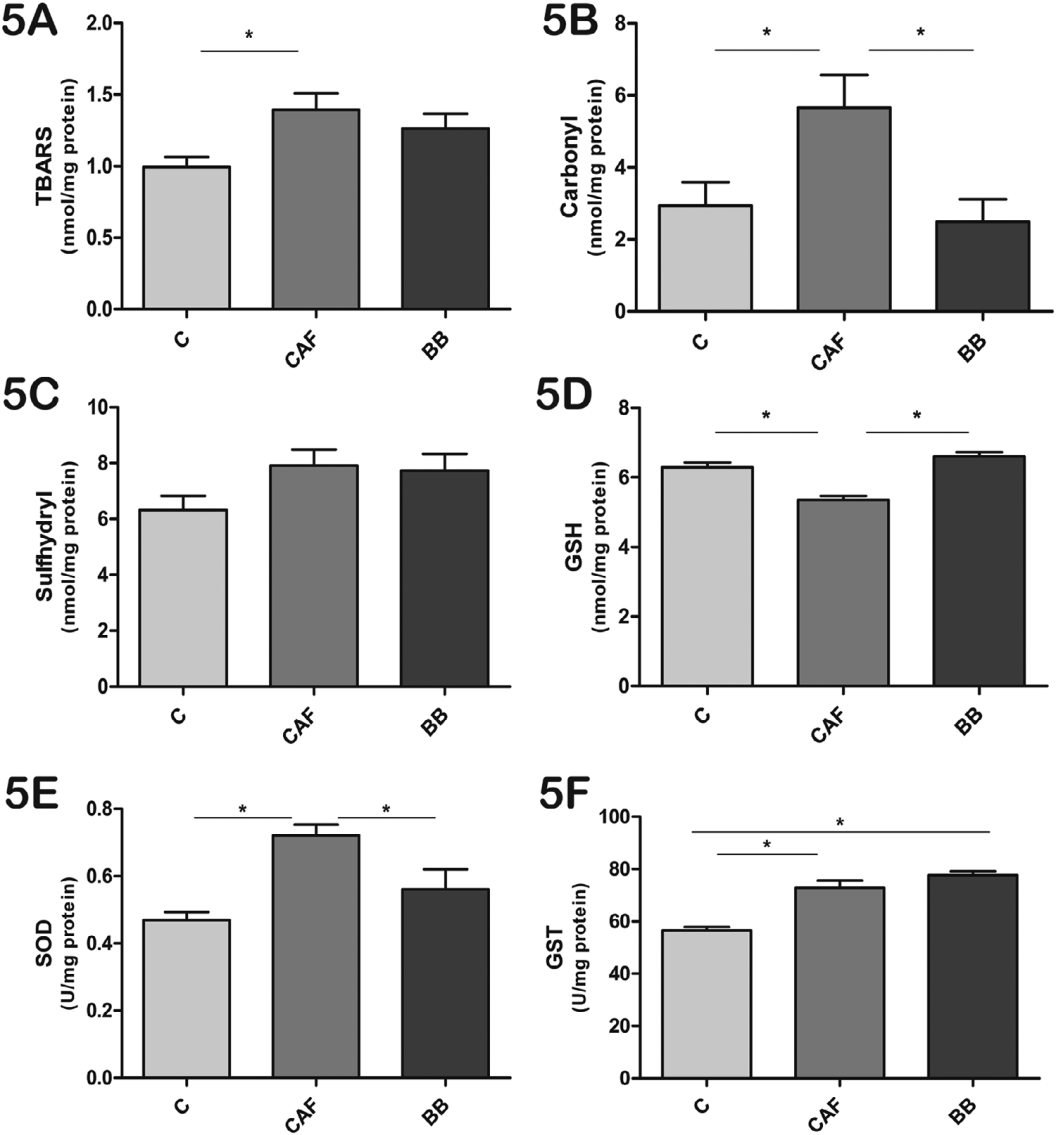
BB supplementation influences oxidative stress and antioxidant markers in the liver. (A) TBARS levels (nmol/mg protein). (B) Carbonyl levels (nmol/mg protein). (C) Sulfhydryl content (nmol/mg protein). (D) GSH concentration (nmol/mg protein). (E) SOD activity (U/mg protein). (F) GST activity (U/mg protein). Values are expressed as mean ± SEM. *n* = 10 in C, *n* = 11 in CAF, and *n* = 10 in BB, representing biological replicates. Significant differences are indicated by ^*^ *p* < 0.05 according to one‐way ANOVA followed by post hoc tests. BB = blueberry, C = control, CAF = cafeteria diet, GSH = glutathione, GST = glutathione‐S‐transferase, SOD = superoxide dismutase, TBARS = thiobarbituric acid reactive species.

In the liver, TBARS levels were higher in the CAF group compared to the C group (Figure 5A); however, BB consumption did not restore TBARS levels to normal. Carbonyl levels were significantly increased in the CAF group compared to both the C and BB mice (Figure 5B), while sulfhydryl content did not differ between the groups (Figure 5C). GSH concentration was lower in CAF mice compared to the BB and C groups (Figure 5D). Similar to the results observed in the brain, SOD activity was also higher in the CAF group compared to both the BB and C groups (Figure 5E); however, GST activity was increased in both the BB and CAF groups compared to the C mice (Figure 5F).

### 3.5 Effects of CAFD and BB Consumption on Obesity‐Related Genes in SAT, VAT, BAT, Liver, Muscle, and Hypothalamus

**FIGURE 6:**
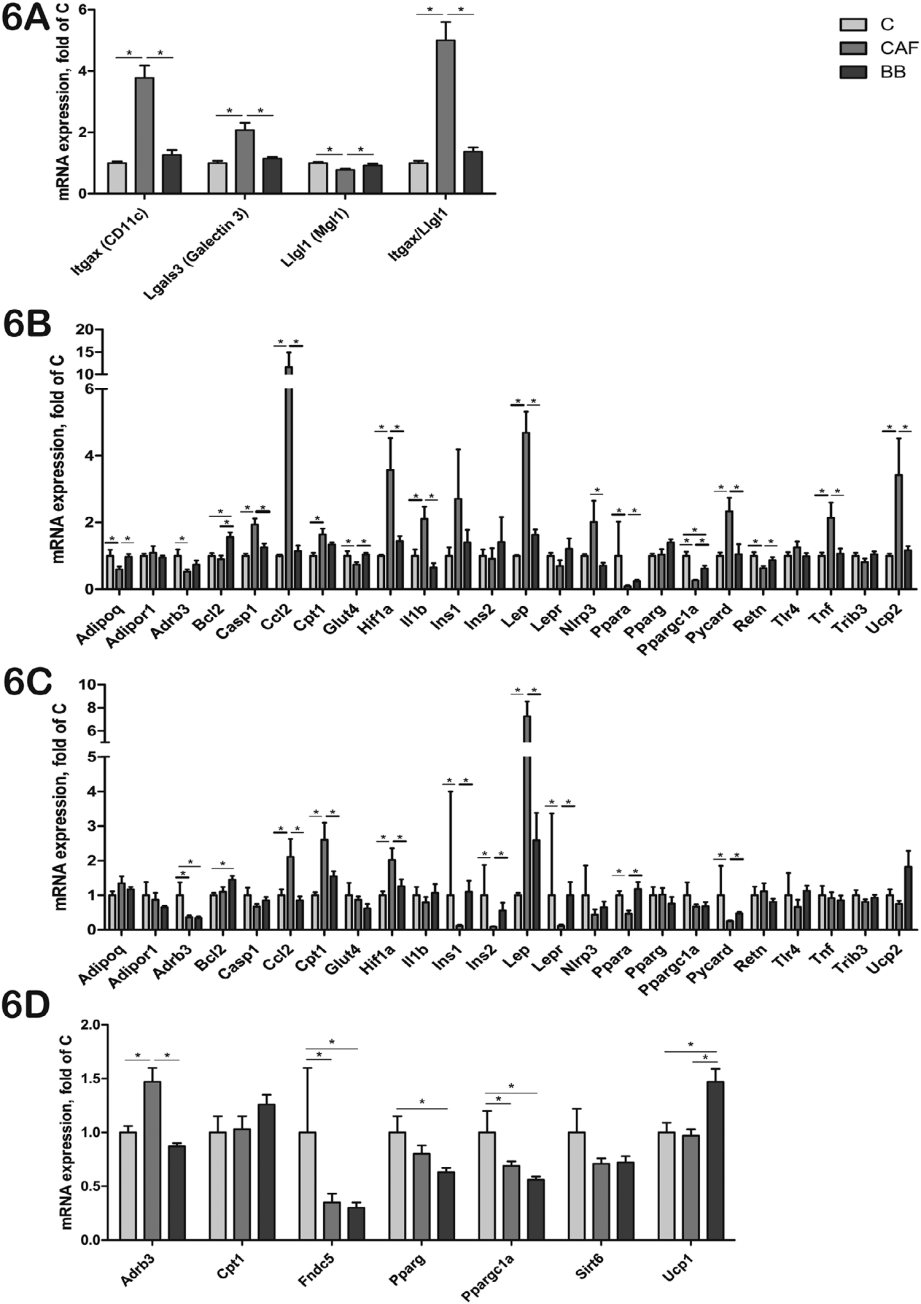
Expressions of genes related to apoptotic, lipid and glucose metabolism, oxidative stress, pro‐inflammatory, and adipocytokine pathways in VAT, SAT, and BAT of C57BL/6 mice. (A) Macrophage markers in VAT. (B) VAT. (C) SAT. D) BAT. Values are expressed as mean ± SEM. *n* = 10 in C, *n* = 11 in CAF, and *n* = 10 in BB, representing biological replicates. Significant differences are indicated by ^*^ *p* < 0.05 according to one‐way ANOVA followed by post hoc tests or Kruskal–Wallis tests, as appropriate. BAT = brown adipose tissue, BB = blueberry, C = control, CAF = cafeteria diet, SAT = subcutaneous adipose tissue, VAT = visceral adipose tissue.

CAFD dysregulated the expression of obesity‐related genes in VAT, SAT, BAT, liver, muscle, and hypothalamus (Table S4). In VAT, the expression of *Itgax* (M1/CD11c+) and *Lgals3* (galectin 3) was upregulated in CAF mice compared to both the C and BB groups (Figure 6A). Moreover, *Llgl1* expression (M2/MGL1+) was downregulated in the CAF group compared to the other two groups (Figure 6A). Consequently, the *Itgax/Llgl1* ratio was significantly higher in CAF mice compared to both the C and BB groups (Figure 6A).

Additionally, in VAT, CAF mice exhibited upregulation of nine other genes (*Casp1*, *Ccl2*, *Cpt1*, *Hif1a*, *Il1b*, *Lep*, *Pycard*, *Tnf*, and *Ucp2*), and downregulation of six genes (*Adipoq*, *Adrb3*, *Glut4*, *Ppara*, *Ppargc1a*, and *Retn*) compared to the C mice (Figure 6B). Interestingly, BB consumption was able to completely or partially restore the expression of these genes to the levels observed in the C group, except for *Cpt1* and *Adrb3*. Notably, *Bcl2* expression was upregulated in BB mice compared to both C and CAF mice. *Nlrp3* expression was increased in the CAF group compared to both the BB and C groups, although the comparison with the C group did not reach statistical significance (Figure 6B).

In SAT, the expressions of *Ccl2*, *Cpt1*, *Hif1a*, and *Lep* were upregulated, while *Adrb3*, *Ins1*, *Ins2*, *Lepr*, *Ppara*, and *Pycard* were downregulated in the CAF group compared to the C group. BB consumption showed positive effects on the expressions of these genes, except for *Adrb3* (Figure 6C). Additionally, *Bcl2* expression was upregulated in the BB group compared to the C group.

Regarding gene expressions in BAT, *Adrb3* was upregulated in the CAF group compared to both the C and BB groups. *Fndc5* and *Ppargc1* expressions were downregulated in both the CAF and BB groups compared to the C group (Figure 6D). *Pparg* expression appeared to be downregulated in both the BB and CAF groups compared to the C group, but only the comparison between the BB and C groups achieved statistical significance. Moreover, *Ucp1* expression was upregulated in BB mice compared to both the C and CAF groups.

**FIGURE 7:**
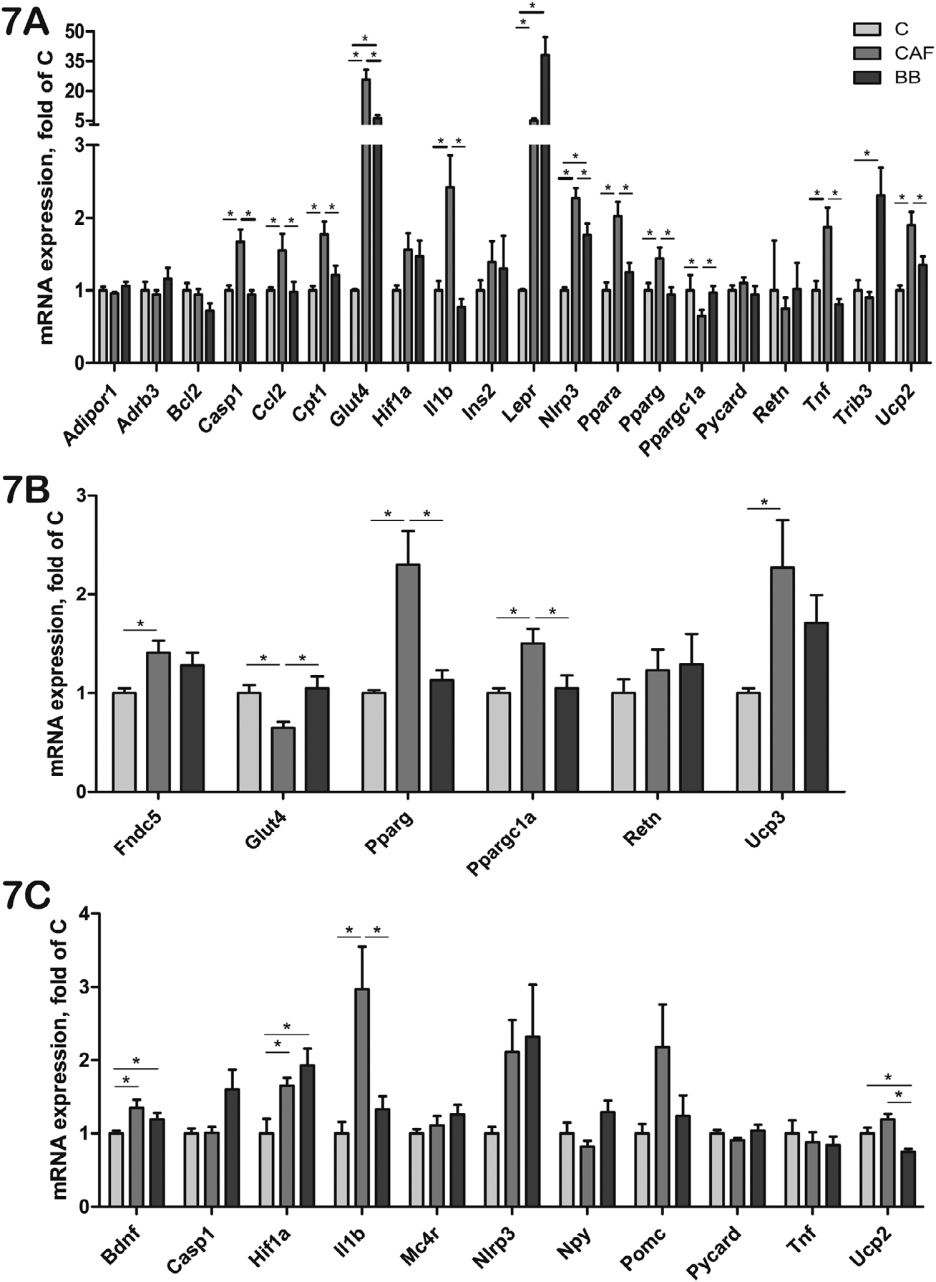
Expressions of genes related to apoptotic, lipid and glucose metabolism, oxidative stress, pro‐inflammatory, and adipocytokine pathways in the liver, skeletal muscle, and hypothalamus of C57BL/6 mice. (A) Liver. (B) Skeletal muscle. (C) Hypothalamus. Values are expressed as mean ± SEM. *n* = 10 in C, *n* = 11 in CAF, and *n* = 10 in BB, representing biological replicates. Significant differences are indicated by ^*^ *p* < 0.05 according to one‐way ANOVA followed by post hoc tests or Kruskal–Wallis tests, as appropriate. BB = blueberry, C = control, CAF = cafeteria diet.

In the liver, CAFD consumption upregulated 11 genes (*Casp1*, *Ccl2*, *Cpt1*, *Glut4*, *Il1b*, *Lepr*, *Nlrp3*, *Ppara*, *Pparg*, *Tnf*, and *Ucp2*), and downregulated one gene (*Ppargc1a*) compared to the C group (Figure 7A). BB consumption exhibited positive effects on the expression of these genes, except for *Glut4* and *Nlrp3*, which showed intermediate expressions between the CAF and C groups, and *LepR*, which had more pronounced expression in the BB group than in the CAF group. Notably, BB consumption also increased the expression of *Trib3* in BB mice compared to the other two groups.

In muscle, *Fndc5*, *Pparg*, *Ppargc1a*, and *Ucp3* were upregulated in CAF mice, while *Glut4* was downregulated in this group compared to the C group (Figure 7B). BB consumption restored *Glut4*, *Pparg*, and *Ppargc1a* expressions to the levels observed in the C group but did not significantly influence *Fndc5* and *Ucp3* expressions. In the hypothalamus, the expressions of *Bdnf*, *Hif1a*, and *Il1b* were upregulated in the CAF mice compared to the C group (Figure 7C). However, BB consumption significantly downregulated only the expression of *Il1b*. Moreover, *Ucp2* expression in the BB group was lower than in both the C and CAF groups.

### 3.6 Correlations Between Gene Expressions in VAT and Obesity‐Related Characteristics

Correlations between significantly dysregulated genes in VAT and obesity‐related characteristics are depicted in Figure S2. Expressions of *Hif1a* and *Lep* were positively correlated with adiponectin circulating levels, HOMA‐IR, 16‐week follow‐up weight, Lee index, and Δ‐weight. Moreover, *Ccl2* expression was positively correlated with 16‐week follow‐up weight, Lee index, and Δ‐weight. Interestingly, expressions of *Ppargc1a* and *Retn* were negatively correlated with adiponectin and irisin circulating levels, as well as 16‐week follow‐up glucose. *Glut4* and *Ppargc1a* were negatively correlated with glucose levels after OGTT. Furthermore, expressions of *Casp1*, *Tnf*, and *Ucp2* were positively correlated with adiponectin circulating levels, while negatively correlated with initial fasting glucose levels. *Adipoq* was negatively correlated with insulin levels, HOMA‐IR, and 16‐week follow‐up weight, but positively correlated with 16‐week follow‐up size. In addition, expressions of *Adrb3* and *Bcl2* were negatively correlated with the Lee index, while expressions of *Adrb3* were positively correlated with 16‐week follow‐up size. Levels of *Bcl2* and *Pycard* were negatively and positively correlated with 16‐week follow‐up weight, respectively. Lastly, the *Itgax/Llgl1* ratio was positively correlated with adiponectin and irisin circulating levels, and with glucose levels after OGTT.

## 4 Discussion

Polyphenols and anthocyanins from BB are suggested to protect cells against oxidative stress and inflammation, thereby reducing the risk of obesity, T2DM, and IR [46, 47, 48]. Among berries, BB has the most diverse profile of anthocyanins [46, 48]. These compounds exhibit anti‐inflammatory, antioxidant, and vasoprotective effects, and can help regulate glucose delivery to insulin‐sensitive tissues and manage whole‐body weight [46, 47, 48]. Here, we demonstrate that supplementing a CAFD with BB (smoothie + fresh‐dried pellets) during a 16‐week follow‐up improved biometric, glycemic, oxidative stress, and hepatic steatosis‐related outcomes in C57BL/6 male mice. Additionally, we investigated the nutrigenomic influences of this diet on a set of obesity‐related pathways in VAT, SAT, BAT, liver, muscle, and hypothalamus from these animals.

Besides its function as a lipid storage site, AT acts as an endocrine organ, synthesizing and releasing numerous adipokines [49]. These molecules play a key role in several physiological and pathophysiological processes, facilitating communication with various organs within the human body [49, 50]. The onset of obesity leads to AT remodeling, reducing the release of anti‐inflammatory adipokines (e.g., adiponectin) and increasing the secretion of pro‐inflammatory adipokines (e.g., leptin) [49, 50]. Subjects with obesity present leptin resistance, affecting energy expenditure and increasing food intake [51, 52]. Studies have shown that mice fed a BB diet exhibited better regulation of circulating leptin levels compared to those on a HFD diet [22, 26], which aligns with our findings. Moreover, here, the expressions of *Lep* in VAT and SAT from the BB group were completely or partially restored to those levels observed in the C group, highlighting BB as a potential fruit for controlling leptin levels in the context of obesity. Furthermore, although the expression of *LepR* in VAT was not influenced by BB consumption, this gene was positively modulated in the SAT and liver of mice in the BB group, which could explain why circulating leptin levels were positively regulated in these animals.

Adiponectin has insulin‐sensitizing, anti‐atherogenic, and anti‐inflammatory effects [53]. Decreased adiponectin serum levels are linked to chronic inflammation, T2DM, obesity, and atherosclerosis [53, 54]. This decrease possibly results from the activation of pathways involved in inflammation, AT hypertrophy, and oxidative stress [53, 54]. Interestingly, in our study, adiponectin serum levels were increased in CAF mice, while the BB group showed intermediate levels of this adipokine compared to the other groups. This result aligns with Vendrame et al. [55], who reported increased levels of serum adiponectin in Zucker rats receiving BB compared to controls. Regarding gene expression, *Adipoq* was downregulated in the VAT of CAF mice, and the BB consumption significantly reduced its expression. Additionally, *Adipoq* expression did not show differences between groups in SAT, while *Adipor1* expression did not differ between groups in VAT, SAT, or liver. Further studies are needed to elucidate the molecular effects of CAFD and BB on adiponectin responses.

Chronic low‐grade inflammation of AT is mechanistically linked to metabolic disease in the presence of overweight and obesity [56, 57]. In particular, inflammation in VAT is intricately intertwined with a crosstalk of several pro‐ and anti‐inflammatory signaling pathways involved in innate and adaptive immune responses to adipocyte hypertrophy, hyperplasia, and hypoxia [56, 57, 58]. The NLRP3 inflammasome‐Asc (Pycard)‐Casp1 complex can be activated by danger‐associated molecules released due to the excess of nutrients [59, 60]. This activation primarily occurs through the TLR4 and contributes to the maturation and secretion of the pro‐inflammatory cytokines IL‐1β and IL‐18 in VAT, leading to AT dysregulation [59, 60]. Recent evidence shows that natural polyphenols can inhibit NLRP3‐inflammasome activation [61]. Fan et al. [62] demonstrated that C57BL/6 mice fed a HFD supplemented with whole red raspberry exhibited higher energy expenditure and were protected against obesity and IR compared to mice that did not receive the fruit. The authors concluded that polyphenols inhibit NLRP3 activation through the inhibition of TLR4/NF‐κB and MAPK pathways [62]. Accordingly, our study demonstrated that expressions of *Nlrp3* (in VAT and liver), *Casp1* (in VAT and liver), and *Il1b* (in VAT, hypothalamus, and liver) were increased in CAF mice, being positively modulated by BB consumption, while *Tlr4* expression was not influenced by CAFD and BB consumption. CAFD increased *Pycard* expression in VAT but decreased it in SAT, with BB consumption restoring its expression to the levels observed in C mice. Moreover, the NLRP3‐inflammasome can be activated by hypoxia, which is at least partially regulated by *Hif1a* [63]. Interestingly, *Hif1a* expression was upregulated in VAT, SAT, and the hypothalamus of CAF mice, while the BB consumption reduced its expression only in VAT and SAT.

Macrophage infiltration into AT is a key inflammatory factor, leading to reduced adipogenesis and lipid storage capacity, adipocyte necrosis and fibrosis, and metabolic disorders, such as IR [64, 65]. M2 (*Llgl1*, MGL1+) macrophages are AT resident cells with an anti‐inflammatory phenotype. The activation of the TLR4/NF‐κB‐mediated inflammatory pathway due to hypercaloric diets can polarize and activate pro‐inflammatory M1 (*Itgax*, CD11c+) macrophages, promoting inflammation [64, 65, 66]. Chemokines, such as the monocyte chemoattractant protein 1 (MCP‐1, encoded by *Ccl2*), play crucial roles in recruiting circulating monocytes (*Lgals3*, Galectin 3+) into AT in the presence of obesity [65, 66]. Several studies have demonstrated that *Ccl2* is upregulated in AT from both humans and mice with obesity [67, 68, 69]. BB anthocyanins appear to inhibit NF‐κB‐related inflammation, decreasing the recruitment of M1 macrophages into AT, and consequently reducing type 1 inflammatory responses [18, 22]. The beneficial effects of monocyte modulation have been demonstrated in clinical and animal studies, linked with decreased oxidative stress after BB supplementation [21, 22, 26, 70]. In line with this, we showed that BB was able to attenuate the effects of CAFD on the expressions of *Itgax*, *Llgl1*, and *Lgals3* in VAT. Moreover, BB markedly reduced CAFD‐induced *Ccl2* upregulation in VAT, SAT, and liver. Consistent with DeFuria et al. [22] results, increased *Itgax/Llgl1* ratio, a hallmark of obesity‐related inflammation, was also attenuated by BB supplementation to the CAFD in our study.

Oxidative stress is another key factor involved in obesity and its complications [19]. The excessive production of ROS is consistently linked to the persistence of obesity‐related low‐grade inflammation and IR [71, 72]. The consumption of foods typical of a Western diet leads to oxidative stress. This disrupts regulatory factors of mitochondrial activity, promotes lipogenesis and differentiation of preadipocytes, and alters the energy balance in hypothalamic neurons that control appetite [71, 72]. We demonstrated that levels of TBARS, sulfhydryl content, SOD, and GST were increased in the brains of CAF mice, while these levels were similar between the BB and C groups. Additionally, GSH levels were lower in CAF mice compared to the C group, but BB consumption did not improve its levels. In the liver, carbonyl, TBARS, SOD, and GST levels were increased in CAF animals, but BB consumption only affected SOD and carbonyl levels. Furthermore, GSH levels were increased in BB mice, indicating an improvement in the liver's antioxidant system.

Overall, these results indicate a positive effect of BB consumption on reducing oxidative stress in the brain and the liver caused by exposure to CAFD. We hypothesize that the increased activity of SOD and GST may occur due to a heightened physiological demand for these enzymes caused by the hypercaloric content of the CAFD [73, 74]. Additionally, the reduced SOD levels in the liver and brain of the BB group may be attributed to the antioxidant properties of BB polyphenols, which directly scavenge ROS, thereby reducing the physiological demand for endogenous enzymes such as SOD [75, 76]. This is consistent with studies indicating that dietary polyphenols modulate antioxidant enzyme activity in response to the oxidative status of specific tissues [75, 76].

Glucotoxicity and lipotoxicity are significant drivers of both oxidative stress and inflammation. Thus, enhancing systemic antioxidant capacity is expected to mitigate the impact of obesity‐related oxidative stress and its associated outcomes [19, 77]. Accordingly, the consumption of berries rich in antioxidant phenolic compounds is associated with increased plasma total antioxidant status in humans [78]. Studies conducted in cellular and animal models of oxidative stress have also demonstrated the positive effects of BB, reinforcing the role of this fruit as an antioxidant dietary supplement [19, 79, 80].

The Bcl2 family of proteins and caspase 9 plays a key role in protecting against oxidative stress‐induced apoptosis [81]. Our study showed that mice fed BB exhibited *Bcl2* upregulation in VAT and SAT compared to both CAF and C groups. Accordingly, another study reported that in HepG2 cells treated with H_2_O_2_, BB extract enhanced the protective effects against oxidative stress through mitochondrial caspase activity and a Bcl‐2‐dependent signal pathway [81, 82]. Moreover, Zhao et al. [82] showed that BB‐derived exosome‐like nanoparticles ameliorated oxidative stress in rotenone‐induced HepG2 cells and HFD‐fed C57BL/6 mice, preventing ROS‐induced apoptosis by inducing *Bcl‐2* expression.

UCPs 1, 2, and 3 are members of an anion‐carrier family localized within the inner mitochondrial membrane, and are key modulators of cellular energy homeostasis [83, 84]. *UCP1* is predominantly expressed in BAT, *UCP3* in the skeletal muscle, and *UCP2* shows ubiquitous expression across various tissues [83, 84]. Dysregulations in *UCP* expressions are associated with impairments of thermogenesis and energy expenditure (*UCP1*), fatty acid metabolism (*UCP2* and *UCP3*), mitochondrial ROS reduction (*UCP1‐3*), negative regulation of insulin secretion (*UCP2*), and cytokine‐induced apoptosis (*UCP2*), all of which are linked to obesity and T2DM [83, 84, 85]. Interestingly, we found that CAFD increased *Ucp2* expression in VAT, the hypothalamus, and the liver, while BB consumption reduced its expression in these tissues. TNF, IL‐1β, glucose, nonesterified fatty acids, and leptin have been shown to induce *UCP2* expression in different cell types [83, 84, 85]. Accordingly, *Tnf* and *Il1b* expressions were increased in VAT and liver of CAF animals, following the expression pattern observed for *Ucp2*, while the BB consumption decreased its expression. Although *UCP2* induction protects cells from oxidative stress, it may also lead to increased cytokine‐induced apoptosis, as well as a reduction in insulin secretion by beta‐cells [84, 85]. Thus, *Ucp2* downregulation by BB supplementation might protect tissues from cytokine damage and improve glucose tolerance, as observed here. *Ucp3* is also induced by higher levels of intramuscular nonesterified fatty acids [83], which are associated with obesity. In line with this, in this study, *Ucp3* was increased in the muscle of the CAF mice, and it was at least partially decreased in the BB group. Additionally, *Ucp1* expression in BAT was upregulated in the BB group, which could possibly lead to increased energy expenditure in this group, contributing to the reduction of the weight of these animals. Consistent with our findings, Liu et al. [86] demonstrated an upregulation of *Ucp1* in BAT of rats fed with BB (HFD + BB) compared to controls.

PPARs play a crucial role in regulating *UCP* expressions [87, 88]. We observed dysregulations in the expressions of *Ppara* (in VAT, SAT, and liver), *Pparg* (liver and muscle), and *Ppargc1a* (in VAT, BAT, liver, and muscle) following CAFD intake. Interestingly, BB partially or completely restored the expression of these genes to levels similar to those of the C group, except for *Ppargc1a* in BAT. Modifications in the expression of PPAR pathway‐related genes may partially explain the alterations in *Ucp* expressions [87, 88]. PPARs also regulate the expression of genes related to lipid metabolism, including *CPT1A*, which encodes an enzyme essential for the carnitine‐shuttle import of long‐chain fatty acids into mitochondria, promoting β‐oxidation [89, 90]. Accordingly, we observed that *Cpt1a* expression was increased in VAT, SAT, and liver of CAF mice. BB supplementation seemed to partially restore its expression. PPARs are also involved in the activation of BAT browning, in part through the induction of irisin, a myokine encoded by the *Fndc5* gene [91]. CAFD dysregulated *Fndc5* expression in both BAT and skeletal muscle, whereas BB supplementation partially restored its expression in muscle. Interestingly, circulating irisin levels were lower in the BB group compared to both the C and CAF groups. This reduction may be attributed to the antioxidant effects of BB, which enhance metabolic efficiency and thereby diminish the need for irisin‐driven adipose tissue browning. Consequently, the lower irisin levels in the BB group likely reflect a decreased physiological demand rather than impaired metabolism [91].

*Glut4* and *Ppargc1a* are indicators of glucose uptake in AT and skeletal muscle, as *Ppargc1a* regulates *Glut4* expression [92]. Notably, our study showed a dysregulation of *Glut4* (in VAT, muscle, and the liver), and *Ins1* and *Ins2* (in SAT) in mice fed a CAFD, which possibly contributed to reduced glucose intake through AKT/MAPK pathways, thus leading to peripheral IR [92]. BB consumption appeared to improve glucose tolerance, as it partially or completely ameliorated *Ins1* and *Ins2* expressions in SAT, as well as *Glut4* in the liver, VAT, and muscle. BB may also improve glucose tolerance by enhancing pancreatic beta‐cell survival and decreasing the expressions of pro‐inflammatory cytokines and oxidative stress [86, 93].

Overall, our findings suggest that dietary BB may confer beneficial effects on key metabolic parameters and hepatic steatosis, largely due to their high polyphenol content (particularly anthocyanins), which possess strong antioxidant and anti‐inflammatory properties [19]. Anthocyanins in BB have been shown to reduce oxidative stress, thereby enhancing glucose tolerance and improving endothelial function [21, 94, 95]. Animal studies have reported decreases in oxidative stress markers, such as TBARS and sulfhydryl oxidation [70, 96]. Additionally, BB intake may mitigate inflammation by inhibiting NF‐κB signaling and reducing the infiltration of pro‐inflammatory immune cells, including M1 macrophages, into adipose tissue [19, 22, 65]. In rodent models, dietary BB also appears to protect against hepatic steatosis by limiting the accumulation of hepatic triglycerides in the liver [96, 97].

Our study should be interpreted with consideration of a few limitations. First, gene expression was assessed only in VAT, SAT, BAT, liver, muscle, and hypothalamus; therefore, the results might differ in other tissues. Second, besides serum leptin, adiponectin, and irisin levels, it was not possible to measure levels of other proteins and lipids due to the insufficient amount of serum collected from the mice. Third, we only analyzed gene expressions in the different tissues because the amount of tissue was not sufficient to extract proteins. Thus, we cannot exclude the possibility of posttranslational modifications of the analyzed genes. Additionally, the limited availability of tissue samples prevented the assessment of oxidative stress markers in VAT and other tissues. Fourth, although the precise BB intake per mouse was not directly measured, monitoring of food leftovers indicated similar consumption across cages. The comparable body weight gain observed among the mice further supports the likelihood of consistent intake of the BB‐supplemented diet. Lastly, the diet effects are applicable only to C57BL/6 mice and may differ in other rodent species, strains, and sexes.

In conclusion, we provided insights into the effects of BB consumption on a CAFD‐induced model of obesity in C57BL/6 mice. BB supplementation was able to control weight gain, leptin and adiponectin levels, and hepatic steatosis, as well as reduce oxidative stress. Regarding glycemic outcomes, BB intake partially decreased hyperglycemia and IR. These effects were accompanied by nutrigenomic modulation of a set of obesity‐related genes involved in apoptotic, lipid and glucose metabolism, oxidative stress, and adipocytokine pathways in VAT, SAT, BAT, liver, muscle, and hypothalamus. Further studies are needed to better elucidate the molecular responses to dietary BB.

## Ethics Statement

This study was approved by the Ethics Committee on Animal Use of Hospital de Clínicas de Porto Alegre, Rio Grande do Sul, Brazil (protocol code: DIPE‐HCPA number 2019‐0546; date of approval: december, 2019).

## Conflicts of Interest

The authors declare no conflicts of interest.

## Supporting information




**Supporting File 1**: mnfr70206‐supp‐0001‐SuppMat.docx

**Supporting File 2**: mnfr70206‐supp‐0002‐TableS1.docx

**Supporting File 3**: mnfr70206‐supp‐0003‐TableS2.docx

S**Supporting File 4**: mnfr70206‐supp‐0004‐TableS3.docx

**Supporting File 5**: mnfr70206‐supp‐0005‐TableS4.docx

**Supporting File 6**: mnfr70206‐supp‐0006‐FigureS1.tif

**Supporting File 7**: mnfr70206‐supp‐0007‐FigureS2.tif

## Data Availability

The data that support the findings of this study are available from the corresponding author upon reasonable request.
