# Supplementary material for: Dietary Blueberry Supplementation Attenuates the Effects of an Ultra‐Processed Food Cafeteria Diet on Weight Gain and Metabolic Parameters, Enhancing Nutrigenomic Profiles in C57BL/6 Mice
Source: Mol Nutr Food Res. 2025 Aug 22;69(21):e70206. doi: 10.1002/mnfr.70206 (PMC12581746; doi:10.1002/mnfr.70206)
Supplement: Supplementary file 1 — Supporting File 1: mnfr70206‐supp‐0001‐SuppMat.docx [file MNFR-69-e70206-s003.docx]

**Supplementary Material 1.** Summary of the pilot experiment performed to determine the most effective method of BB supplementation.

A pilot study was conducted prior to the main experiment to identify and standardize the most effective method of BB supplementation. Ten male C57BL/6 mice were housed in pairs, under controlled conditions (12-h light/dark cycle, 22 ± 2°C, 40–60% humidity, with an air exhaust system). Over a four-week period, five supplementation methods were tested: (1) fresh BB blended with condensed milk and water; (2) fresh BB blended with water; (3) freeze-dried BB pellets mixed with water; (4) a smoothie made from freeze-dried BB pellets, water, and condensed milk; and (5) solid freeze-dried BB pellet pieces. BB intake was evaluated every two days by weighing the amount offered and the leftovers. The highest acceptance was observed with the smoothie prepared from freeze-dried BB pellets, water, and condensed milk, as well as with the solid freeze-dried BB pellet pieces. Consequently, these two methods were selected for BB administration in the main study.
