# Supplementary material for: Dietary Blueberry Supplementation Attenuates the Effects of an Ultra‐Processed Food Cafeteria Diet on Weight Gain and Metabolic Parameters, Enhancing Nutrigenomic Profiles in C57BL/6 Mice
Source: Mol Nutr Food Res. 2025 Aug 22;69(21):e70206. doi: 10.1002/mnfr.70206 (PMC12581746; doi:10.1002/mnfr.70206)
Supplement: Supplementary file 2 — Supporting File 2: mnfr70206‐supp‐0002‐TableS1.docx [file MNFR-69-e70206-s007.docx]

**Supplementary Table 1. Oligonucleotide primers used for quantitative real-time PCR (qPCR).**

| **Gene** | **F and R primer sequences** |
| --- | --- |
| *Actb* (reference) | F 5’ AGATGACCCAGATCATGTTTGAGA 3’ |
|  | R 5’ CACAGCCTGGATGGCTACGT 3’ |
| *Cyclofilin* (reference) | F 5’ GGGTTCCTCCTTTCACAGAA 3’ |
|  | R 5’ GATGCCAGGACCTGTATGCT 3’ |
| *Eif2a* (reference) | F 5’ GCTGGGACGCCTAACCTACA 3’ |
|  | R 5’ GGATGAACGATTTCAAACATGCT 3’ |
| *Rplp0* (reference) | F 5’ TGGGCATCACCACGAAAAT 3’ |
|  | R 5’ ATCAGCTGCACATCACTCAGAATT 3’ |
| *Ywhaz* (reference) | F 5’ TGGTGCCATGACCCATCTT 3’ |
|  | R 5’ TCTGGGAGCTGAGGCAAAAT 3’ |
| *Adipoq* | F 5’ GACACCAAAAGGGCTCAGGAT 3’ |
|  | R 5’ TGGGCAGGATTAAGAGGAACA 3’ |
| *Adipoqr1* | F 5’ GTATAAGGTCTGGGAGGGACGTT 3’ |
|  | R 5’ AGTCGTTGTCTTTCAGCCAGTCA 3’ |
| *Adrb3* | F 5’ CGCTCCAGAACCGATGACTAG 3’ |
|  | R 5’ GACGGTGAAACCCATTTGGTA 3’ |
| *Bcl2* | F 5’ AAGGGCTTCACACCCAAATCT 3’ |
|  | R 5’ TTCTACGTCTGCTTGGCTTTGA 3’ |
| *Bdnf* | F 5’ TTCGGCCCAACGAAGAAA 3’ |
|  | R 5’ AGCATCACCCGGGAAGTG 3’ |
| *Casp1* | F 5’ CGTACACGTCTTGCCCTCATTA 3’ |
|  | R 5’ CCAACCCTCGGAGAAAGATG 3’ |
| *Ccl2* | F 5’ CTGAAGCCAGCTCTCTCTTCCT 3’ |
|  | R 5’ CAGGCCCAGAAGCATGACA 3’ |
| *Cpt1* | F 5’ GGGCCGGTTGCTGATG 3’ |
|  | R 5’ TCAGGGCTAGAGAACTTGGAAGA 3’ |
| *Fndc5* | F 5’ TCATGTGGGCAGGTGTTATAGC 3’ |
|  | R 5’ GGGCTCGTTGTCCTTGATGAT 3’ |
| *Glut4* | F 5’ CAGCCTACGCCACCATAGGA 3’ |
|  | R 5’ CTAAGAGCACCGAGACCAACGT 3’ |
| *Hif1a* | F 5’ GCGGGCACCGATTCG 3’ |
|  | R 5’ CGTTCAGAACTCATCCTATTTTTCTTC 3’ |
| *Il1b* | F 5’ TGACAGTGATGAGAATGACCTGTTC 3’ |
|  | R 5’ TTGGAAGCAGCCCTTCATCT 3’ |
| *Ins1* | F 5’ TCA GAG ACC ATC AGC AAG 3’ |
|  | R 5’ GGGACCACAAAGATGCTGTT 3’ |
| *Ins2* | F 5’ GGAGCGTGGCTTCTTCTACA 3’ |
|  | R 5’ CAGTGCCAAGGTCTGAAG GT 3’ |
| *Itgax* | F 5’ TGGGCCTGTCCCTTGCT 3’ |
|  | R 5’ ACAGTAGGACCACAAGCCAACA 3’ |
| *Lep* | F 5’ CACACACGCAGTCGGTATCC 3’ |
|  | R 5’ GGTGAAGCCCAGGAATGAAG 3’ |
| *Lepr* | F 5’ TGGAAGCCCCTGACGAAA 3’ |
|  | R 5’ CATTGTGGGCAGTACGATGCT 3’ |
| *Lgals3* | F 5’ CCAACGCAAACAGGATTGTTC 3’ |
|  | R 5’ GGGTTAAAGTGGAAGGCAACAT 3’ |
| *Llgl1* | F 5’ GACTCGCTCTCCGGTGTTGT 3’ |
|  | R 5’ GTCGCATCTCGAAGGAACGT 3’ |
| *Mc4r* | F 5’ TAGCCTGGCTGTGGCAGAT 3’ |
|  | R 5’ CGATGGTTTCCGACCCATT 3’ |
| *Nlrp3* | F 5’ GACCATCGGCCGGACTAAA 3’ |
|  | R 5’ CGTCCTCGGGCTCAAACA 3’ |
| *Npy* | F 5’ CCGCTCTGCGACACTACATC 3’ |
|  | R 5’ GGGCTGGATCTCTTGCCATA 3’ |
| *Pomc* | F 5’ TGAACATCTTTGTCCCCAGAGA 3’ |
|  | R 5’ TCCCGCTATCTTTCCAACATG 3’ |
| *Pparα* | F 5’ CAAGAGGCTGTGTGACCTAGTGA 3’ |
|  | R 5’ GACTAAATTTTGCATGTGTGCATCT 3’ |
| *Pparg* | F 5’ GCCCACCAACTTCGGAATC 3’ |
|  | R 5’ TGCGAGTGGTCTTCCATCAC 3’ |
| *Ppargc1a* | F 5’ CTGCCATTGTTAAGACCGAGAA 3’ |
|  | R 5’ AGGGACGTCTTTGTGGCTTTT 3’ |
| *Pycard* | F 5’ GATGCTTTGCATGGCAGTGT 3’ |
|  | R 5’ CTGGTGGTCTCTGCACGAACT 3’ |
| *Retn* | F 5’ TTCCTGATGTCGGTCAGTTGAG 3’ |
|  | R 5’ TCCCCGTCCCTGTCAACA 3’ |
| *Sirt6* | F 5’ TGCCCCTTGCCACTAAGC 3’ |
|  | R 5’ TGGGTTGCAGGTTGACAATG 3’ |
| *Tlr4* | F 5’ GCTAAGTGCCGAGTCTGAGTGTAA 3’ |
|  | R 5’ TGCAGCCTTTCAGAAACACATT 3’ |
| *Tnf* | F 5’ GACCCTCACACTCAGATCATCTTCT 3’ |
|  | R 5’ CCACTTGGTGGTTTGCTACGA 3’ |
| *Trib3* | F 5’ GGAAGATGCCTGCGTGATG 3’ |
|  | R 5’ AGGGCACGCATGCTTGTC 3’ |
| *Ucp1* | F 5’ CGATGTCCATGTACACCAAGGA 3’ |
|  | R 5’ AAAAGAAGCCACAAACCCTTTG 3’ |
| *Ucp2* | F 5’ GCCTCTGGAAAGGGACTTCTC 3’ |
|  | R 5’ ACCAGCTCAGCACAGTTGACA 3’ |
| *Ucp3* | F 5’ GGGACCATGGTTGGACTTCA 3’ |
|  | R 5’ GCCCCCAGGAACTTCACAA 3’ |
