# Supplementary material for: Dietary Blueberry Supplementation Attenuates the Effects of an Ultra‐Processed Food Cafeteria Diet on Weight Gain and Metabolic Parameters, Enhancing Nutrigenomic Profiles in C57BL/6 Mice
Source: Mol Nutr Food Res. 2025 Aug 22;69(21):e70206. doi: 10.1002/mnfr.70206 (PMC12581746; doi:10.1002/mnfr.70206)
Supplement: Supplementary file 3 — Supporting File 3: mnfr70206‐supp‐0003‐TableS2.docx [file MNFR-69-e70206-s001.docx]

**Supplementary Table 2. cDNA concentrations and reference genes used to assess gene expression in the different tissues of interest.**

| **Gene** | **cDNA (ng)** | **Reference gene** |
| --- | --- | --- |
| ***SAT*** |  |  |
| *Adipoq* | 100 | *Cyclofilin* |
| *Adipoqr1* | 50 | *Cyclofilin* |
| *Adrb3* | 50 | *Cyclofilin* |
| *Bcl2* | 50 | *Ywhaz* |
| *Casp1* | 50 | *Cyclofilin* |
| *Ccl2* | 200 | *Cyclofilin* |
| *Cpt1* | 50 | *Cyclofilin* |
| *Glut4* | 100 | *Ywhaz* |
| *Hif1a* | 100 | *Cyclofilin* |
| *Il1b* | 200 | *Cyclofilin* |
| *Ins1* | 200 | *Cyclofilin* |
| *Ins2* | 100 | *Cyclofilin* |
| *Lep* | 100 | *Cyclofilin* |
| *Lepr* | 200 | *Cyclofilin* |
| *Nlrp3* | 100 | *Cyclofilin* |
| *Pparα* | 200 | *Cyclofilin* |
| *Pparg* | 100 | *Cyclofilin* |
| *Ppargc1a* | 100 | *Cyclofilin* |
| *Pycard* | 50 | *Cyclofilin* |
| *Retn* | 100 | *Cyclofilin* |
| *Tlr4* | 100 | *Cyclofilin* |
| *Tnf* | 100 | *Ywhaz* |
| *Trib3* | 100 | *Ywhaz* |
| *Ucp2* | 100 | *Cyclofilin* |
| ***VAT*** |  |  |
| *Adipoq* | 100 | *Cyclofilin* |
| *Adipoqr1* | 25 | *Cyclofilin* |
| *Adrb3* | 200 | *Cyclofilin* |
| *Bcl2* | 50 | *Cyclofilin* |
| *Casp1* | 50 | *Cyclofilin* |
| *Ccl2* | 100 | *Cyclofilin* |
| *Cpt1* | 25 | *Cyclofilin* |
| *Glut4* | 200 | *Cyclofilin* |
| *Hif1a* | 25 | *Cyclofilin* |
| *Il1b* | 50 | *Cyclofilin* |
| *Ins1* | 200 | *Cyclofilin* |
| *Ins2* | 50 | *Cyclofilin* |
| *Itgax* | 100 | *Cyclofilin* |
| *Lep* | 25 | *Cyclofilin* |
| *Lepr* | 200 | *Cyclofilin* |
| *Lgals3* | 100 | *Cyclofilin* |
| *Llgl1* | 100 | *Cyclofilin* |
| *Nlrp3* | 100 | *Cyclofilin* |
| *Pparα* | 200 | *Cyclofilin* |
| *Pparg* | 6.3 | *Cyclofilin* |
| *Ppargc1a* | 12.5 | *Cyclofilin* |
| *Pycard* | 100 | *Cyclofilin* |
| *Retn* | 100 | *Cyclofilin* |
| *Tlr4* | 25 | *Cyclofilin* |
| *Tnf* | 100 | *Cyclofilin* |
| *Trib3* | 25 | *Cyclofilin* |
| *Ucp2* | 100 | *Cyclofilin* |
| ***BAT*** |  |  |
| *Adrb3* | 100 | *Cyclofilin* |
| *Cpt1* | 100 | *Cyclofilin* |
| *Fndc5* | 100 | *Cyclofilin* |
| *Pparg* | 100 | *Cyclofilin* |
| *Ppargc1a* | 100 | *Cyclofilin* |
| *Sirt6* | 100 | *Cyclofilin* |
| *Ucp1* | 100 | *Cyclofilin* |
| ***Liver*** |  |  |
| *Adipoqr1* | 100 | *Cyclofilin* |
| *Adrb3* | 200 | *Rplp0* |
| *Bcl2* | 50 | *Eif2a* |
| *Casp1* | 200 | *Eif2a* |
| *Ccl2* | 200 | *Cyclofilin* |
| *Cpt1* | 200 | *Rplp0* |
| *Glut4* | 200 | *Rplp0* |
| *Hif1a* | 200 | *Eif2a* |
| *Il1b* | 200 | *Eif2a* |
| *Ins2* | 200 | *Eif2a* |
| *Lepr* | 200 | *Eif2a* |
| *Nlrp3* | 200 | *Actb* |
| *Pparα* | 200 | *Eif2a* |
| *Pparg* | 200 | *Cyclofilin* |
| *Ppargc1a* | 200 | *Actb* |
| *Pycard* | 100 | *Eif2a* |
| *Retn* | 200 | *Eif2a* |
| *Tnf* | 200 | *Eif2a* |
| *Trib3* | 200 | *Cyclofilin* |
| *Ucp2* | 200 | *Cyclofilin* |
| ***Muscle*** |  |  |
| *Fndc5* | 100 | *Ywhaz* |
| *Glut4* | 100 | *Ywhaz* |
| *Pparg* | 100 | *Ywhaz* |
| *Ppargc1a* | 100 | *Ywhaz* |
| *Retn* | 200 | *Ywhaz* |
| *Ucp3* | 100 | *Ywhaz* |
| ***Hypothalamus*** |  |  |
| *Bdnf* | 100 | *Rplp0* |
| *Casp1* | 200 | *Rplp0* |
| *Hif1a* | 100 | *Actb* |
| *Il1b* | 400 | *Rplp0* |
| *Mc4r* | 50 | *Rplp0* |
| *Nlrp3* | 200 | *Rplp0* |
| *Npy* | 100 | *Rplp0* |
| *Pomc* | 400 | *Rplp0* |
| *Pycard* | 200 | *Rplp0* |
| *Tnf* | 400 | *Rplp0* |
| *Ucp2* | 100 | *Rplp0* |
