# Supplementary material for: Dietary Blueberry Supplementation Attenuates the Effects of an Ultra‐Processed Food Cafeteria Diet on Weight Gain and Metabolic Parameters, Enhancing Nutrigenomic Profiles in C57BL/6 Mice
Source: Mol Nutr Food Res. 2025 Aug 22;69(21):e70206. doi: 10.1002/mnfr.70206 (PMC12581746; doi:10.1002/mnfr.70206)
Supplement: Supplementary file 4 — SSupporting File 4: mnfr70206‐supp‐0004‐TableS3.docx [file MNFR-69-e70206-s006.docx]

**Supplementary Table 3. Measurement of oxidative stress and antioxidant makers in the brain and liver.**

| **Variables** | **C (n = 10)** | **CAF (n = 12)** | **BB (n = 10)** | **P** |
| --- | --- | --- | --- | --- |
| ***Brain*** |  |  |  |  |
| TBARS (nmol/mg protein) | 1.1 ± 0.5 ^a^ | 1.5 ± 0.5 ^b^ | 1.0 ± 0.6 ^a^ | <0.0001 |
| Carbonyl (nmol/mg protein) | 1.2 ± 0.3 | 2.2 ± 1.0 | 1.5 ± 0.4 | 0.991 |
| Sulfhydryl (nmol/mg protein) | 7.6 ± 0.3 ^a^ | 9.6 ± 0.7 ^b^ | 7.7 ± 0.5 ^a^ | 0.016 |
| GST (U/mg protein) | 46.6 ± 1.4 ^a^ | 52.2 ± 1.5 ^b^ | 43.6 ± 1.3 ^a^ | <0.001 |
| SOD (U/mg protein) | 1.4 ± 0.1 ^a^ | 2.2 ± 0.1 ^b^ | 1.3 ± 0.1 ^a^ | <0.0001 |
| GSH (nmol/mg protein) | 10.8 ± 0.3 ^a^ | 9.2 ± 0.2 ^b^ | 9.5 ± 0.2 ^b^ | <0.0001 |
| ***Liver*** |  |  |  |  |
| TBARS (nmol/mg protein) | 1.0 ± 0.1 ^a^ | 1.4 ± 0.1 ^b^ | 1.3 ± 0.1 ^ab^ | 0.021 |
| Carbonyl (nmol/mg protein) | 2.9 ± 0.6 ^a^ | 5.6 ± 0.9 ^b^ | 2.5 ± 0.6 ^a^ | 0.013 |
| Sulfhydryl (nmol/mg protein) | 6.3 ± 0.5 | 7.9 ± 0.6 | 7.7 ± 0.6 | 0.108 |
| GST (U/mg protein) | 56.6 ± 1.3 ^a^ | 72.9 ± 2.8 ^b^ | 77.7 ± 1.5 ^b^ | < 0.0001 |
| SOD (U/mg protein) | 0.5 ± 0.1 ^a^ | 0.7 ± 0.1 ^b^ | 0.6 ± 0.1 ^a^ | < 0.0001 |
| GSH (nmol/mg protein) | 6.3 ± 0.1 ^a^ | 5.4 ± 0.1 ^b^ | 6.6 ± 0.1 ^a^ | < 0.0001 |

Values are expressed as mean ± SEM. C = standard diet; CAF = cafeteria diet; BB = cafeteria diet + blueberries; TBARS: thiobarbituric acid reactive species; GST: glutathione-S-transferase; SOD: superoxide reductase; GSH: glutathione. P values were obtained with One-Way ANOVA followed by post-hoc or with Kruskal-Wallis tests, as appropriate. Variables with equal letters do not differ significantly in the statistical tests; those with different letters were statistically different.
