# Supplementary material for: Dietary Blueberry Supplementation Attenuates the Effects of an Ultra‐Processed Food Cafeteria Diet on Weight Gain and Metabolic Parameters, Enhancing Nutrigenomic Profiles in C57BL/6 Mice
Source: Mol Nutr Food Res. 2025 Aug 22;69(21):e70206. doi: 10.1002/mnfr.70206 (PMC12581746; doi:10.1002/mnfr.70206)
Supplement: Supplementary file 5 — Supporting File 5: mnfr70206‐supp‐0005‐TableS4.docx [file MNFR-69-e70206-s002.docx]

**Supplementary Table 4. Relative gene expressions by qPCR in SAT, VAT, BAT, liver, muscle, and hypothalamus of C, CAF and BB mice after 16 weeks of follow-up.**

| **Genes** | **C (n=10)** | **CAF (n=11)** | **BB (n=10)** | **P*** |
| --- | --- | --- | --- | --- |
| ***SAT*** | | | | |
| *Adipoq* | 1.00 ± 0.11 | 1.35 ± 0.20 | 1.17 ± 0.07 | 0.248* |
| *Adipoqr1* | 1.00 ± 0.38 | 0.87 ± 0.20 | 0.65 ± 0.05 | 0.802** |
| *Adrb3* | 1.00 ± 0.37 ^a^ | 0.36 ± 0.06 ^b^ | 0.34 ± 0.05 ^b^ | < 0.0001* |
| *Bcl2* | 1.00 ± 0.07 ^a^ | 1.10 ± 0.14 ^a^ | 1.45 ± 0.11 ^b^ | 0.028* |
| *Casp1* | 1.00 ± 0.22 | 0.67 ± 0.08 | 0.85 ± 0.10 | 0.174* |
| *Ccl2* | 1.00 ± 0.17 ^a^ | 2.11 ± 0.52 ^b^ | 0.85 ± 0.12 ^a^ | 0.034** |
| *Cpt1* | 1.00 ± 0.09 ^a^ | 2.60 ± 0.50 ^b^ | 1.55 ± 0.14 ^a^ | 0.012* |
| *Glut4* | 1.00 ± 0.36 | 0.87 ± 0.10 | 0.62 ± 0.13 | 0.445* |
| *Hif1a* | 1.00 ± 0.11 ^a^ | 2.02 ± 0.34 ^b^ | 1.26 ± 0.20 ^a^ | 0.014** |
| *Il1b* | 1.00 ± 0.24 | 0.79 ± 0.16 | 1.07 ± 0.25 | 0.592* |
| *Ins1* | 1.00 ± 3.00 ^a^ | 0.12 ± 0.02 ^b^ | 1.10 ± 0.32 ^a^ | 0.031* |
| *Ins2* | 1.00 ± 0.88 ^a^ | 0.09 ± 0.01 ^b^ | 0.56 ± 0.22 ^a^ | 0.026** |
| *Lep* | 1.00 ± 0.07 ^a^ | 7.26 ± 1.28 ^b^ | 2.59 ± 0.79 ^a^ | < 0.0001* |
| *Lepr* | 1.00 ± 2.37 ^a^ | 0.12 ± 0.03 ^b^ | 1.00 ± 0.38 ^a^ | 0.036** |
| *Nlrp3* | 1.00 ± 0.86 | 0.44 ± 0.15 | 0.65 ± 0.17 | 0.212** |
| *Pparα* | 1.00 ± 0.12 ^a^ | 0.46 ± 0.10 ^b^ | 1.18 ± 0.20 ^a^ | 0.019* |
| *Pparg* | 1.00 ± 0.24 | 1.02 ± 0.19 | 0.76 ± 0.19 | 0.936* |
| *Ppargc1a* | 1.00 ± 0.37 | 0.67 ± 0.07 | 0.68 ± 0.12 | 0.313* |
| *Pycard* | 1.00 ± 0.85 ^a^ | 0.25 ± 0.03 ^b^ | 0.47 ± 0.05 ^a^ | 0.002** |
| *Retn* | 1.00 ± 0.25 | 1.11 ± 0.24 | 0.81 ± 0.09 | 0.824** |
| *Tlr4* | 1.00 ± 0.64 | 0.66 ± 0.21 | 1.13 ± 0.15 | 0.262* |
| *Tnf* | 1.00 ± 0.27 | 0.92 ± 0.17 | 0.85 ± 0.14 | 0.876* |
| *Trib3* | 1.00 ± 0.15 | 0.81 ± 0.07 | 0.93 ± 0.08 | 0.324* |
| *Ucp2* | 1.00 ± 0.17 | 0.75 ± 0.09 | 1.82 ± 0.46 | 0.349** |
| ***VAT*** | | | | |
| *Adipoq* | 1.00 ± 0.18 ^a^ | 0.60 ± 0.08 ^b^ | 0.97 ± 0.09 ^a^ | 0.031* |
| *Adipoqr1* | 1.00 ± 0.07 | 1.09 ± 0.20 | 0.95 ± 0.06 | 0.751* |
| *Adrb3* | 1.00 ± 0.19 ^a^ | 0.53 ± 0.07 ^b^ | 0.74 ± 0.12 ^ab^ | 0.032** |
| *Bcl2* | 1.00 ± 0.08 ^a^ | 0.90 ± 0.11 ^a^ | 1.56 ± 0.14 ^b^ | 0.004* |
| *Casp1* | 1.00 ± 0.07 ^a^ | 1.94 ± 0.18 ^b^ | 1.25 ± 0.12 ^a^ | < 0.0001* |
| *Ccl2* | 1.00 ± 0.03 ^a^ | 11.6 ± 3.27 ^b^ | 1.14 ± 0.17 ^a^ | < 0.0001** |
| *Cpt1* | 1.00 ± 0.09 ^a^ | 1.64 ± 0.18 ^b^ | 1.34 ± 0.06 ^ab^ | 0.027* |
| *Glut4* | 1.00 ± 0.14 ^a^ | 0.74 ± 0.07 ^b^ | 1.04 ± 0.05 ^a^ | 0.030* |
| *Hif1a* | 1.00 ± 0.03 ^a^ | 3.57 ± 0.96 ^b^ | 1.44 ± 0.15 ^c^ | < 0.0001** |
| *Il1b* | 1.00 ± 0.19 ^a^ | 2.11 ± 0.36 ^b^ | 0.65 ± 0.13 ^a^ | < 0.001* |
| *Ins1* | 1.00 ± 0.25 | 2.71 ± 1.48 | 1.40 ± 0.38 | 0.493* |
| *Ins2* | 1.00 ± 0.19 | 0.91 ± 0.32 | 1.41 ± 0.75 | 0.967** |
| *Itgax* | 1.00 ± 0.06 ^a^ | 3.78 ± 0.40 ^b^ | 1.27 ± 0.16 ^a^ | < 0.0001* |
| *Lep* | 1.00 ± 0.02 ^a^ | 4.69 ± 0.63 ^b^ | 1.63 ± 0.16 ^a^ | < 0.0001** |
| *Lepr* | 1.00 ± 0.09 | 0.70 ± 0.17 | 1.21 ± 0.31 | 0.307** |
| *Llgl1* | 1.00 ± 0.04 ^a^ | 0.78 ± 0.04 ^b^ | 0.92 ± 0.06 ^ab^ | 0.019* |
| *Lgals3* | 1.00 ± 0.07 ^a^ | 2.08 ± 0.23 ^b^ | 1.15 ± 0.05 ^a^ | < 0.0001* |
| *Nlrp3* | 1.00 ± 0.06 ^ab^ | 2.01 ± 0.64 ^b^ | 0.71 ± 0.08 ^a^ | 0.007** |
| *Pparα* | 1.00 ± 1.02 ^a^ | 0.09 ± 0.03 ^b^ | 0.24 ± 0.05 ^a^ | 0.007** |
| *Pparg* | 1.00 ± 0.07 | 1.04 ± 0.16 | 1.40 ± 0.10 | 0.205* |
| *Ppargc1a* | 1.00 ± 0.12 ^a^ | 0.27 ± 0.02 ^b^ | 0.62 ± 0.09 ^c^ | < 0.0001* |
| *Pycard* | 1.00 ± 0.10 ^a^ | 2.33 ± 0.41 ^b^ | 1.05 ± 0.30 ^a^ | 0.012* |
| *Retn* | 1.00 ± 0.11 ^a^ | 0.63 ± 0.07 ^b^ | 0.87 ± 0.09 ^a^ | 0.029* |
| *Tlr4* | 1.00 ± 0.11 | 1.25 ± 0.18 | 0.99 ± 0.09 | 0.584** |
| *Tnf* | 1.00 ± 0.10 ^a^ | 2.14 ± 0.46 ^b^ | 1.07 ± 0.15 ^a^ | 0.032* |
| *Trib3* | 1.00 ± 0.09 | 0.81 ± 0.11 | 1.05 ± 0.08 | 0.202* |
| *Ucp2* | 1.00 ± 0.07 ^a^ | 3.42 ± 1.10 ^b^ | 1.17 ± 0.12 ^a^ | < 0.0001** |
| ***BAT*** | | | | |
| *Adrb3* | 1.00 ± 0.06 ^a^ | 1.47 ± 0.13 ^b^ | 0.87 ± 0.03 ^a^ | < 0.001* |
| *Cpt1* | 1.00 ± 0.15 | 1.03 ± 0.12 | 1.26 ± 0.09 | 0.313* |
| *Fndc5* | 1.00 ± 0.60 ^a^ | 0.35 ± 0.08 ^b^ | 0.30 ± 0.05 ^b^ | 0.010** |
| *Pparg* | 1.00 ± 0.15 ^a^ | 0.80 ± 0.08 ^ab^ | 0.63 ± 0.04 ^b^ | 0.025* |
| *Ppargc1a* | 1.00 ± 0.20 ^a^ | 0.69 ± 0.04 ^b^ | 0.56 ± 0.03 ^b^ | 0.004* |
| *Sirt6* | 1.00 ± 0.22 | 0.71 ± 0.05 | 0.72 ± 0.06 | 0.113* |
| *Ucp1* | 1.00 ± 0.09 ^a^ | 0.97 ± 0.06 ^a^ | 1.47 ± 0.12 ^b^ | < 0.001* |
| ***Liver*** | | | | |
| *Adipoqr1* | 1.00 ± 0.05 | 0.96 ± 0.02 | 1.06 ± 0.06 | 0.382* |
| *Adrb3* | 1.00 ± 0.12 | 0.94 ± 0.06 | 1.16 ± 0.15 | 0.432* |
| *Bcl2* | 1.00 ± 0.10 | 0.94 ± 0.08 | 0.72 ± 0.10 | 0.109* |
| *Casp1* | 1.00 ± 0.07 ^a^ | 1.67 ± 0.17 ^b^ | 0.94 ± 0.06 ^a^ | 0.002* |
| *Ccl2* | 1.00 ± 0.04 ^a^ | 1.55 ± 0.23 ^b^ | 0.98 ± 0.14 ^a^ | 0.018** |
| *Cpt1* | 1.00 ± 0.06 ^a^ | 1.77 ± 0.18 ^b^ | 1.21 ± 0.13 ^c^ | 0.003* |
| *Glut4* | 1.00 ± 0.02 ^a^ | 25.65 ± 5.04 ^b^ | 6.18 ± 1.56 ^c^ | < 0.001* |
| *Hif1a* | 1.00 ± 0.07 | 1.56 ± 0.23 | 1.47 ± 0.22 | 0.141* |
| *Il1b* | 1.00 ± 0.13 ^a^ | 2.42 ± 0.44 ^b^ | 0.77 ± 0.11 ^a^ | 0.002* |
| *Ins2* | 1.00 ± 0.14 | 1.39 ± 0.29 | 1.30 ± 0.45 | 0.709* |
| *Lepr* | 1.00 ± 0.02 ^a^ | 5.11 ± 0.91 ^b^ | 38.0 ± 9.09 ^c^ | 0.005* |
| *Nlrp3* | 1.00 ± 0.04 ^a^ | 2.27 ± 0.14 ^b^ | 1.76 ± 0.16 ^c^ | < 0.0001* |
| *Pparα* | 1.00 ± 0.11 ^a^ | 2.02 ± 0.20 ^b^ | 1.25 ± 0.13 ^a^ | < 0.001* |
| *Pparg* | 1.00 ± 0.10 ^a^ | 1.44 ± 0.15 ^b^ | 0.94 ± 0.10 ^a^ | 0.021* |
| *Ppargc1a* | 1.00 ± 0.21 ^a^ | 0.65 ± 0.08 ^b^ | 0.97 ± 0.09 ^a^ | 0.031* |
| *Pycard* | 1.00 ± 0.07 | 1.10 ± 0.08 | 0.94 ± 0.12 | 0.444** |
| *Retn* | 1.00 ± 0.69 | 0.75 ± 0.15 | 1.02 ± 0.36 | 0.762** |
| *Tnf* | 1.00 ± 0.13 ^a^ | 1.87 ± 0.27 ^b^ | 0.81 ± 0.07 ^a^ | < 0.0001* |
| *Trib3* | 1.00 ± 0.14 ^a^ | 0.90 ± 0.08 ^a^ | 2.31 ± 0.38 ^b^ | 0.003** |
| *Ucp2* | 1.00 ± 0.07 ^a^ | 1.90 ± 0.18 ^b^ | 1.35 ± 0.12 ^a^ | < 0.0001* |
| ***Muscle*** | | | | |
| *Fndc5* | 1.00 ± 0.05 ^a^ | 1.41 ± 0.12 ^b^ | 1.28 ± 0.13 ^ab^ | 0.044** |
| *Glut4* | 1.00 ± 0.08 ^a^ | 0.65 ± 0.06 ^b^ | 1.05 ± 0.12 ^a^ | 0.010* |
| *Pparg* | 1.00 ± 0.03 ^a^ | 2.30 ± 0.34 ^b^ | 1.13 ± 0.10 ^a^ | < 0.0001** |
| *Ppargc1a* | 1.00 ± 0.05 ^a^ | 1.50 ± 0.15 ^b^ | 1.05 ± 0.13 ^a^ | 0.016* |
| *Retn* | 1.00 ± 0.14 | 1.23 ± 0.21 | 1.29 ± 0.31 | 0.697* |
| *Ucp3* | 1.00 ± 0.05 ^a^ | 2.27 ± 0.48 ^b^ | 1.71 ± 0.28 ^b^ | 0.017** |
| ***Hypothalamus*** | | | | |
| *Bdnf* | 1.00 ± 0.04 ^a^ | 1.35 ± 0.11 ^b^ | 1.19 ± 0.09 ^ab^ | 0.034* |
| *Casp1* | 1.00 ± 0.07 | 1.01 ± 0.08 | 1.60 ± 0.27 | 0.178** |
| *Hif1a* | 1.00 ± 0.02 ^a^ | 1.65 ± 0.11 ^b^ | 1.93 ± 0.23 ^b^ | 0.005* |
| *Il1b* | 1.00 ± 0.16 ^a^ | 2.97 ± 0.58 ^b^ | 1.33 ± 0.18 ^a^ | 0.002* |
| *Mc4r* | 1.00 ± 0.06 | 1.11 ± 0.13 | 1.26 ± 0.13 | 0.284** |
| *Nlrp3* | 1.00 ± 0.09 | 2.11 ± 0.44 | 2.32 ± 0.71 | 0.193** |
| *Npy* | 1.00 ± 0.15 | 0.82 ± 0.08 | 1.29 ± 0.16 | 0.064* |
| *Pomc* | 1.00 ± 0.13 | 2.18 ± 0.58 | 1.24 ± 0.28 | 0.126* |
| *Pycard* | 1.00 ± 0.05 | 0.91 ± 0.03 | 1.04 ± 0.08 | 0.280** |
| *Tnf* | 1.00 ± 0.18 | 0.88 ± 0.12 | 0.84 ± 0.12 | 0.718* |
| *Ucp2* | 1.00 ± 0.08 ^a^ | 1.19 ± 0.08 ^a^ | 0.75 ± 0.04 ^b^ | <0.0001* |

Values are expressed as mean ± SEM. *Values obtained by One-Way ANOVA followed by post-hoc tests. **Values obtained by Kruskal-Wallis Test. C = standard diet; CAF = cafeteria diet; BB: cafeteria diet + blueberries; SAT = subcutaneous adipose tissue; VAT = visceral adipose tissue; BAT: brown adipose tissue.
