# Supplementary figures and images for: Dietary Blueberry Supplementation Attenuates the Effects of an Ultra‐Processed Food Cafeteria Diet on Weight Gain and Metabolic Parameters, Enhancing Nutrigenomic Profiles in C57BL/6 Mice
Source: Mol Nutr Food Res. 2025 Aug 22;69(21):e70206. doi: 10.1002/mnfr.70206 (PMC12581746; doi:10.1002/mnfr.70206)

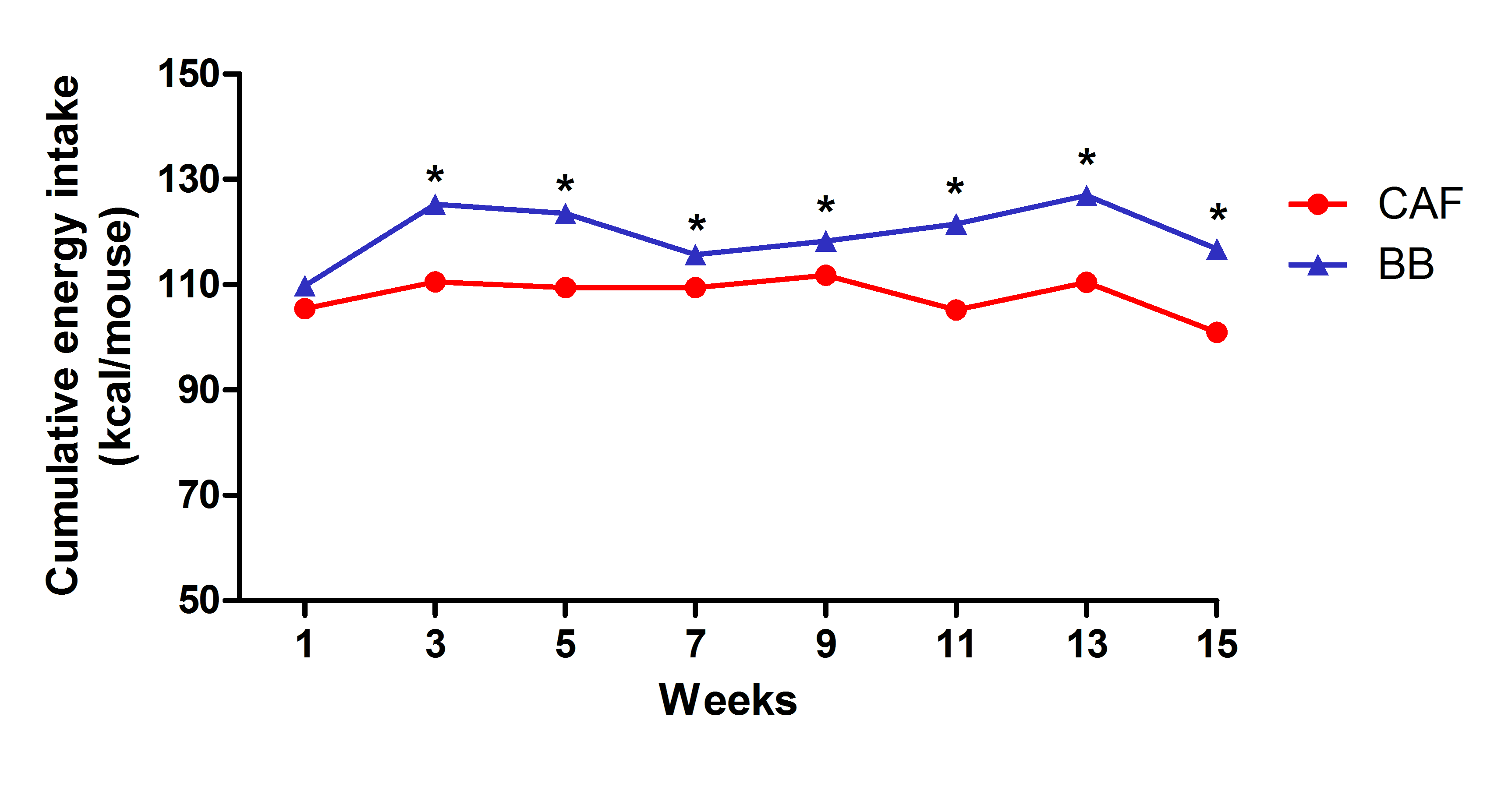

Supplement: Supplementary file 6 — Supporting File 6: mnfr70206‐supp‐0006‐FigureS1.tif [file MNFR-69-e70206-s004.tif]

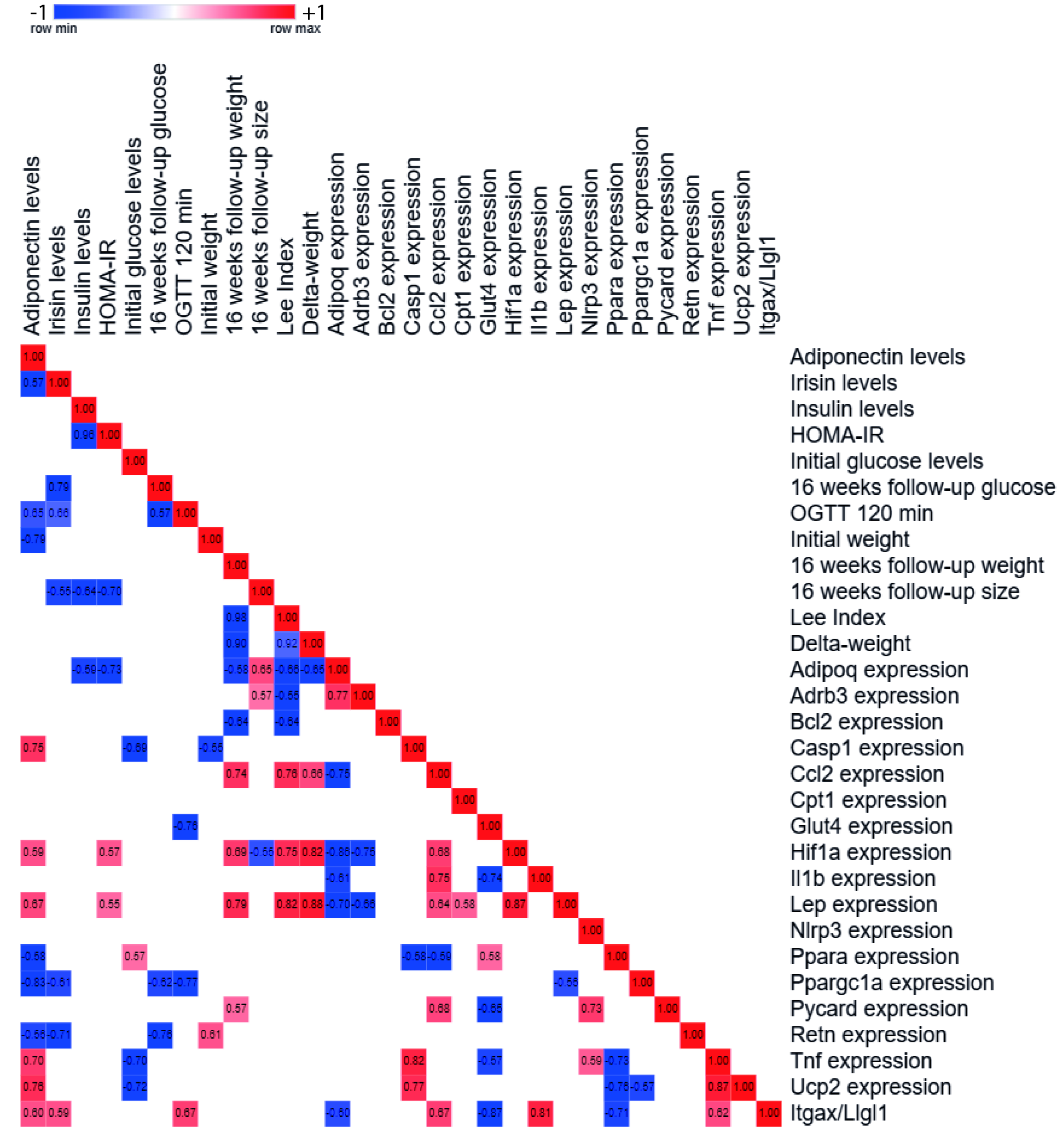

Supplement: Supplementary file 7 — Supporting File 7: mnfr70206‐supp‐0007‐FigureS2.tif [file MNFR-69-e70206-s005.tif]
